# Author Correction: Common variants in Alzheimer’s disease and risk stratification by polygenic risk scores

**DOI:** 10.1038/s41467-023-36192-x

**Published:** 2023-02-09

**Authors:** Itziar de Rojas, Sonia Moreno-Grau, Niccolo Tesi, Benjamin Grenier-Boley, Victor Andrade, Iris E. Jansen, Nancy L. Pedersen, Najada Stringa, Anna Zettergren, Isabel Hernández, Laura Montrreal, Carmen Antúnez, Anna Antonell, Rick M. Tankard, Joshua C. Bis, Rebecca Sims, Céline Bellenguez, Inés Quintela, Antonio González-Perez, Miguel Calero, Emilio Franco-Macías, Juan Macías, Rafael Blesa, Laura Cervera-Carles, Manuel Menéndez-González, Ana Frank-García, Jose Luís Royo, Fermin Moreno, Raquel Huerto Vilas, Miquel Baquero, Mónica Diez-Fairen, Carmen Lage, Sebastián García-Madrona, Pablo García-González, Emilio Alarcón-Martín, Sergi Valero, Oscar Sotolongo-Grau, Abbe Ullgren, Adam C. Naj, Afina W. Lemstra, Alba Benaque, Alba Pérez-Cordón, Alberto Benussi, Alberto Rábano, Alessandro Padovani, Alessio Squassina, Alexandre de Mendonça, Alfonso Arias Pastor, Almar A. L. Kok, Alun Meggy, Ana Belén Pastor, Ana Espinosa, Anaïs Corma-Gómez, Angel Martín Montes, Ángela Sanabria, Anita L. DeStefano, Anja Schneider, Annakaisa Haapasalo, Anne Kinhult Ståhlbom, Anne Tybjærg-Hansen, Annette M. Hartmann, Annika Spottke, Arturo Corbatón-Anchuelo, Arvid Rongve, Barbara Borroni, Beatrice Arosio, Benedetta Nacmias, Børge G. Nordestgaard, Brian W. Kunkle, Camille Charbonnier, Carla Abdelnour, Carlo Masullo, Carmen Martínez Rodríguez, Carmen Muñoz-Fernandez, Carole Dufouil, Caroline Graff, Catarina B. Ferreira, Caterina Chillotti, Chandra A. Reynolds, Chiara Fenoglio, Christine Van Broeckhoven, Christopher Clark, Claudia Pisanu, Claudia L. Satizabal, Clive Holmes, Dolores Buiza-Rueda, Dag Aarsland, Dan Rujescu, Daniel Alcolea, Daniela Galimberti, David Wallon, Davide Seripa, Edna Grünblatt, Efthimios Dardiotis, Emrah Düzel, Elio Scarpini, Elisa Conti, Elisa Rubino, Ellen Gelpi, Eloy Rodriguez-Rodriguez, Emmanuelle Duron, Eric Boerwinkle, Evelyn Ferri, Fabrizio Tagliavini, Fahri Küçükali, Florence Pasquier, Florentino Sanchez-Garcia, Francesca Mangialasche, Frank Jessen, Gaël Nicolas, Geir Selbæk, Gemma Ortega, Geneviève Chêne, Georgios Hadjigeorgiou, Giacomina Rossi, Gianfranco Spalletta, Giorgio Giaccone, Giulia Grande, Giuliano Binetti, Goran Papenberg, Harald Hampel, Henri Bailly, Henrik Zetterberg, Hilkka Soininen, Ida K. Karlsson, Ignacio Alvarez, Ildebrando Appollonio, Ina Giegling, Ingmar Skoog, Ingvild Saltvedt, Innocenzo Rainero, Irene Rosas Allende, Jakub Hort, Janine Diehl-Schmid, Jasper Van Dongen, Jean-Sebastien Vidal, Jenni Lehtisalo, Jens Wiltfang, Jesper Qvist Thomassen, Johannes Kornhuber, Jonathan L. Haines, Jonathan Vogelgsang, Juan A. Pineda, Juan Fortea, Julius Popp, Jürgen Deckert, Katharina Buerger, Kevin Morgan, Klaus Fließbach, Kristel Sleegers, Laura Molina-Porcel, Lena Kilander, Leonie Weinhold, Lindsay A. Farrer, Li-San Wang, Luca Kleineidam, Lucia Farotti, Lucilla Parnetti, Lucio Tremolizzo, Lucrezia Hausner, Luisa Benussi, Lutz Froelich, M. Arfan Ikram, M. Candida Deniz-Naranjo, Magda Tsolaki, Maitée Rosende-Roca, Malin Löwenmark, Marc Hulsman, Marco Spallazzi, Margaret A. Pericak-Vance, Margaret Esiri, María Bernal Sánchez-Arjona, Maria Carolina Dalmasso, María Teresa Martínez-Larrad, Marina Arcaro, Markus M. Nöthen, Marta Fernández-Fuertes, Martin Dichgans, Martin Ingelsson, Martin J. Herrmann, Martin Scherer, Martin Vyhnalek, Mary H. Kosmidis, Mary Yannakoulia, Matthias Schmid, Michael Ewers, Michael T. Heneka, Michael Wagner, Michela Scamosci, Miia Kivipelto, Mikko Hiltunen, Miren Zulaica, Montserrat Alegret, Myriam Fornage, Natalia Roberto, Natasja M. van Schoor, Nazib M. Seidu, Nerisa Banaj, Nicola J. Armstrong, Nikolaos Scarmeas, Norbert Scherbaum, Oliver Goldhardt, Oliver Hanon, Oliver Peters, Olivia Anna Skrobot, Olivier Quenez, Ondrej Lerch, Paola Bossù, Paolo Caffarra, Paolo Dionigi Rossi, Paraskevi Sakka, Patrizia Mecocci, Per Hoffmann, Peter A. Holmans, Peter Fischer, Peter Riederer, Qiong Yang, Rachel Marshall, Rajesh N. Kalaria, Richard Mayeux, Rik Vandenberghe, Roberta Cecchetti, Roberta Ghidoni, Ruth Frikke-Schmidt, Sandro Sorbi, Sara Hägg, Sebastiaan Engelborghs, Seppo Helisalmi, Sigrid Botne Sando, Silke Kern, Silvana Archetti, Silvia Boschi, Silvia Fostinelli, Silvia Gil, Silvia Mendoza, Simon Mead, Simona Ciccone, Srdjan Djurovic, Stefanie Heilmann-Heimbach, Steffi Riedel-Heller, Teemu Kuulasmaa, Teodoro del Ser, Thibaud Lebouvier, Thomas Polak, Tiia Ngandu, Timo Grimmer, Valentina Bessi, Valentina Escott-Price, Vilmantas Giedraitis, Vincent Deramecourt, Wolfgang Maier, Xueqiu Jian, Yolande A. L. Pijnenburg, EADB contributors, A. David Smith, Aldo Saenz, Alessandra Bizzarro, Alessandra Lauria, Alessandro Vacca, Alina Solomon, Anna Anastasiou, Anna Richardson, Anne Boland, Anne Koivisto, Antonio Daniele, Antonio Greco, Arnaoutoglou Marianthi, Bernadette McGuinness, Bertrand Fin, Camilla Ferrari, Carlo Custodero, Carlo Ferrarese, Carlos Ingino, Carlos Mangone, Carlos Reyes Toso, Carmen Martínez, Carolina Cuesta, Carolina Muchnik, Catharine Joachim, Cecilia Ortiz, Céline Besse, Charlotte Johansson, Chiara Paola Zoia, Christoph Laske, Costas Anastasiou, Dana Lis Palacio, Daniel G. Politis, Daniel Janowitz, David Craig, David M. Mann, David Neary, Deckert Jürgen, Delphine Daian, Diyana Belezhanska, Eduardo Kohler, Eduardo M. Castaño, Effrosyni Koutsouraki, Elena Chipi, Ellen De Roeck, Emanuele Costantini, Emma R. L. C. Vardy, Fabrizio Piras, Fausto Roveta, Federica Piras, Federico Ariel Prestia, Francesca Assogna, Francesca Salani, Gessica Sala, Giordano Lacidogna, Gisela Novack, Gordon Wilcock, Håkan Thonberg, Heike Kölsch, Heike Weber, Henning Boecker, Ignacio Etchepareborda, Irene Piaceri, Jaakko Tuomilehto, Jaana Lindström, Jan Laczo, Janet Johnston, Jean-François Deleuze, Jenny Harris, Jonathan M. Schott, Josef Priller, Juan Ignacio Bacha, Julie Snowden, Julieta Lisso, Kalina Yonkova Mihova, Latchezar Traykov, Laura Morelli, Luis Ignacio Brusco, Malik Rainer, Mari Takalo, Maria Bjerke, Maria Del Zompo, Maria Serpente, Mariana Sanchez Abalos, Mario Rios, Markku Peltonen, Martin J. Herrman, Matias Kohler, Matias Rojo, Matthew Jones, Michela Orsini, Nancy Medel, Natividad Olivar, Nick C. Fox, Nicola Salvadori, Nigel M. Hooper, Pablo Galeano, Patricia Solis, Patrizia Bastiani, Peter Passmore, Reinhard Heun, Riitta Antikainen, Robert Olaso, Robert Perneczky, Sandra Germani, Sara López-García, Seth Love, Shima Mehrabian, Silvia Bagnoli, Silvia Kochen, Simona Andreoni, Stefan Teipel, Stephen Todd, Stuart Pickering-Brown, Teemu Natunen, Thomas Tegos, Tiina Laatikainen, Timo Strandberg, Tuomo M. Polvikoski, Vaclav Matoska, Valentina Ciullo, Valeria Cores, Vincenzo Solfrizzi, Viviana Lisetti, Zulma Sevillano, The GR@ACE study group, C. Abdelnour, N. Aguilera, E. Alarcon, M. Alegret, A. Benaque, M. Boada, M. Buendia, P. Cañabate, A. Carracedo, A. Corbatón-Anchuelo, I. de Rojas, S. Diego, A. Espinosa, A. Gailhajenet, P. García-González, S. Gil, M. Guitart, A. González-Pérez, I. Hernández, M. Ibarria, A. Lafuente, J. Macias, O. Maroñas, E. Martín, M. T. Martínez, M. Marquié, A. Mauleón, L. Montrreal, S. Moreno-Grau, M. Moreno, A. Orellana, G. Ortega, A. Pancho, E. Pelejá, A. Pérez-Cordon, J. A. Pineda, S. Preckler, I. Quintela, L. M. Real, M. Rosende-Roca, A. Ruiz, M. E. Sáez, A. Sanabria, M. Serrano-Rios, O. Sotolongo-Grau, L. Tárraga, S. Valero, L. Vargas, DEGESCO consortium, A. D. Adarmes-Gómez, E. Alarcón-Martín, M. D. Alonso, I. Álvarez, V. Álvarez, G. Amer-Ferrer, M. Antequera, C. Antúnez, M. Baquero, M. Bernal, R. Blesa, D. Buiza-Rueda, M. J. Bullido, J. A. Burguera, M. Calero, F. Carrillo, M. Carrión-Claro, M. J. Casajeros, J. Clarimón, J. M. Cruz-Gamero, M. M. de Pancorbo, I. de Rojas, T. del Ser, M. Diez-Fairen, R. Escuela, L. Garrote-Espina, J. Fortea, E. Franco-Macías, A. Frank-García, J. M. García-Alberca, S. Garcia Madrona, G. Garcia-Ribas, P. Gómez-Garre, S. Hevilla, S. Jesús, M. A. Labrador Espinosa, C. Lage, A. Legaz, A. Lleó, A. Lopez de Munain, S. López-García, D. Macias-García, S. Manzanares, M. Marín, J. Marín-Muñoz, T. Marín, M. Marquié, A. Martín Montes, B. Martínez, C. Martínez, V. Martínez, P. Martínez-Lage Álvarez, M. Medina, M. Mendioroz Iriarte, M. Menéndez-González, P. Mir, J. L. Molinuevo, P. Pastor, J. Pérez Tur, T. Periñán-Tocino, R. Pineda-Sanchez, G. Piñol-Ripoll, A. Rábano, D. Real de Asúa, S. Rodrigo, E. Rodríguez-Rodríguez, J. L. Royo, R. Sanchez del Valle Díaz, P. Sánchez-Juan, I. Sastre, M. P. Vicente, R. Vigo-Ortega, L. Vivancos, IGAP (ADGC, CHARGE, EADI, GERAD), C. Macleod, C. McCracken, Carol Brayne, Catherine Bresner, Detelina Grozeva, Eftychia Bellou, Ewen W. Sommerville, F. Matthews, Ganna Leonenko, Georgina Menzies, Gill Windle, Janet Harwood, Judith Phillips, K. Bennett, Lauren Luckuck, Linda Clare, Robert Woods, Salha Saad, Vanessa Burholt, PGC-ALZ consortia, Arvid Rongve, Patrick Gavin Kehoe, Guillermo Garcia-Ribas, Pascual Sánchez-Juan, Pau Pastor, Jordi Pérez-Tur, Gerard Piñol-Ripoll, Adolfo Lopez de Munain, Jose María García-Alberca, María J. Bullido, Victoria Álvarez, Alberto Lleó, Luis M. Real, Pablo Mir, Miguel Medina, Philip Scheltens, Henne Holstege, Marta Marquié, María Eugenia Sáez, Ángel Carracedo, Philippe Amouyel, Gerard D. Schellenberg, Julie Williams, Sudha Seshadri, Cornelia M. van Duijn, Karen A. Mather, Raquel Sánchez-Valle, Manuel Serrano-Ríos, Adelina Orellana, Lluís Tárraga, Kaj Blennow, Martijn Huisman, Ole A. Andreassen, Danielle Posthuma, Jordi Clarimón, Mercè Boada, Wiesje M. van der Flier, Alfredo Ramirez, Jean-Charles Lambert, Sven J. van der Lee, Agustín Ruiz

**Affiliations:** 1 Research Center and Memory clinic Fundació ACE, Institut Català de Neurociències Aplicades, Universitat Internacional de Catalunya, Barcelona, Spain; 2 CIBERNED, Network Center for Biomedical Research in Neurodegenerative Diseases, National Institute of Health Carlos III, Madrid, Spain; 3 Alzheimer Center Amsterdam, Department of Neurology, Amsterdam Neuroscience, Vrije Universiteit Amsterdam, Amsterdam UMC, Amsterdam, The Netherlands; 4 Section Genomics of Neurodegenerative Diseases and Aging, Department of Clinical Genetics, Vrije Universiteit Amsterdam, Amsterdam UMC, Amsterdam, The Netherlands; 5 Delft Bioinformatics Lab, Delft Univeristy of Technology, Delft, The Netherlands; 6 Univ. Lille, Inserm, Institut Pasteur de Lille, CHU Lille, U1167-Labex DISTALZ-RID-AGE-Risk Factors and Molecular Determinants of Aging-Related Diseases, Lille, France; 7 Division of Neurogenetics and Molecular Psychiatry, Department of Psychiatry and Psychotherapy, University of Cologne, Medical Faculty, Cologne, Germany; 8 Department of Neurodegenerative diseases and Geriatric Psychiatry, University Clinic Bonn, Bonn, Germany; 9 Department of Complex Trait Genetics, Center for Neurogenomics and Cognitive Research, Amsterdam Neuroscience, VU University, Amsterdam, The Netherlands; 10 Department of Medical Epidemiology and Biostatistics, Karolinska Institutet, Stockholm, Sweden; 11 Amsterdam UMC-Vrije Universiteit Amsterdam, Department of Epidemiology and Data Science, Amsterdam Public Health Research Institute, Amsterdam, The Netherlands; 12 Neuropsychiatric Epidemiology Unit, Department of Psychiatry and Neurochemistry, Institute of Neuroscience and Physiology, Sahlgrenska Academy, Centre for Ageing and Health (AgeCap), University of Gothenburg, Gothenburg, Sweden; 13 Unidad de Demencias, Hospital Clínico Universitario Virgen de la Arrixaca, Murcia, Spain; 14 Alzheimer’s disease and other cognitive disorders unit. Service of Neurology, Hospital Clínic of Barcelona. Institut d’Investigacions Biomèdiques August Pi i Sunyer, University of Barcelona, Barcelona, Spain; 15 Mathematics and Statistics, Murdoch University, Perth, WA Australia; 16 Cardiovascular Health Research Unit, Department of Medicine, University of Washington, Seattle, WA USA; 17 Division of Psychological Medicine and Clinial Neurosciences, MRC Centre for Neuropsychiatric Genetics and Genomics, Cardiff University, Cardiff, UK; 18 Grupo de Medicina Xenómica, Centro Nacional de Genotipado (CEGEN-PRB3-ISCIII), Universidade de Santiago de Compostela, Santiago de Compostela, Spain; 19 CAEBI, Centro Andaluz de Estudios Bioinformáticos, Sevilla, Spain; 20 UFIEC, Instituto de Salud Carlos III, Madrid, Spain; 21 CIEN Foundation/Queen Sofia Foundation Alzheimer Center, Madrid, Spain; 22 Unidad de Demencias, Servicio de Neurología y Neurofisiología, Instituto de Biomedicina de Sevilla (IBiS), Hospital Universitario Virgen del Rocío/CSIC/Universidad de Sevilla, Sevilla, Spain; 23 Unidad Clínica de Enfermedades Infecciosas y Microbiología, Hospital Universitario de Valme, Sevilla, Spain; 24 Department of Neurology, II B Sant Pau, Hospital de la Santa Creu i Sant Pau, Universitat Autònoma de Barcelona, Barcelona, Spain; 25 Servicio de Neurología, Hospital Universitario Central de Asturias, Oviedo, Spain; 26 Instituto de Investigación Sanitaria del Principado de Asturias (ISPA), Oviedo, Spain; 27 Departamento de Medicina, Universidad de Oviedo, Oviedo, Spain; 28 Department of Neurology, La Paz University Hospital, Instituto de Investigación Sanitaria del Hospital Universitario La Paz, IdiPAZ, Madrid, Spain; 29 Hospital La Paz Institute for Health Research, IdiPAZ, Madrid, Spain; 30 Universidad Autónoma de Madrid, Madrid, Spain; 31 Departamento de Especialidades Quirúrgicas, Bioquímicas e Inmunología, School of Medicine, University of Málaga, Málaga, Spain; 32 Department of Neurology, Hospital Universitario Donostia, San Sebastian, Spain; 33 Neurosciences Area, Instituto Biodonostia, San Sebastian, Spain; 34 Unitat Trastorns Cognitius, Hospital Universitari Santa Maria de Lleida, Lleida, Spain; 35 Institut de Recerca Biomedica de Lleida (IRBLLeida), Lleida, Spain; 36 Servei de Neurologia, Hospital Universitari i Politècnic La Fe, Valencia, Spain; 37 Fundació Docència i Recerca MútuaTerrassa, Terrassa, Barcelona, Spain; 38 Memory Disorders Unit, Department of Neurology, Hospital Universitari Mutua de Terrassa, Terrassa, Barcelona, Spain; 39 Neurology Service, Marqués de Valdecilla University Hospital (University of Cantabria and IDIVAL), Santander, Spain; 40 Hospital Universitario Ramon y Cajal, IRYCIS, Madrid, Spain; 41 Karolinska Institutet, Center for Alzheimer Research, Department NVS, Division of Neurogeriatrics, Stockholm, Sweden; 42 Unit for Hereditary Dementias, Theme Aging, Karolinska University Hospital-Solna, Stockholm, Sweden; 43 Department of Biostatistics, Epidemiology and Informatics, University of Pennsylvania Perelman School of Medicine, Philadelphia, PA USA; 44 Penn Neurodegeneration Genomics Center, Department of Pathology and Laboratory Medicine, University of Pennsylvania Perelman School of Medicine, Philadelphia, PA USA; 45 Centre for Neurodegenerative Disorders, Department of Clinical and Experimental Sciences, University of Brescia, Brescia, Italy; 46 BT-CIEN, Madrid, Spain; 47 Department of Biomedical Sciences, Section of Neuroscience and Clinical Pharmacology, University of Cagliari, Cagliari, Italy; 48 Faculty of Medicine, University of Lisbon, Lisbon, Portugal; 49 Amsterdam UMC, Vrije Universiteit Amsterdam, Department of Psychiatry, Amsterdam Public Health Research Institute, Amsterdam, The Netherlands; 50 UK Dementia Research Institute at Cardiff, Cardiff University, Cardiff, UK; 51 Department of Neurology, La Paz University Hospital, Madrid, Spain; 52 Department of Neurology, Boston University School of Medicine, Boston, MA USA; 53 Department of Biostatistics, Boston University School of Public Health, Boston, MA USA; 54 German Center for Neurodegenerative Diseases (DZNE), Bonn, Germany; 55 A.I Virtanen Institute for Molecular Sciences, University of Eastern Finland, Kuopio, Finland; 56 Department of Clinical Biochemistry, Rigshospitalet, Copenhagen, Denmark; 57 Department of Clinical Medicine, Faculty of Health and Medical Sciences, University of Copenhagen, Copenhagen, Denmark; 58 Martin-Luther-University Halle-Wittenberg, University Clinic and Outpatient Clinic for Psychiatry, Psychotherapy and Psychosomatics, Halle (Saale), Germany; 59 Department of Neurology, University of Bonn, Bonn, Germany; 60 Instituto de Investigación Sanitaria, Hospital Clínico San Carlos (IdISSC), Madrid, Spain; 61 Spanish Biomedical Research Centre in Diabetes and Associated Metabolic Disorders (CIBERDEM), Madrid, Spain; 62 Haugesund Hospital, Helse Fonna, Department of Research and Innovation, Haugesund, Norway; 63 University of Bergen, Institute of Clinical Medicine (K1), Bergen, Norway; 64 Department of Clinical Sciences and Community Health, University of Milan, Milan, Italy; 65 Geriatic Unit, Fondazione Cà Granda, IRCCS Ospedale Maggiore Policlinico, Milan, Italy; 66 Department of Neuroscience, Psychology, Drug Research and Child Health University of Florence, Florence, Italy; 67 IRCCS Fondazione Don Carlo Gnocchi, Florence, Italy; 68 Department of Clinical Biochemistry, Herlev Gentofte Hospital, Herlev, Denmark; 69 Dr. John T. Macdonald Foundation Department of Human Genetics, University of Miami Miller School of Medicine, Miami, FL USA; 70 John P. Hussman Institute for Human Genomics, University of Miami Miller School of Medicine, Miami, FL USA; 71 Normandie Univ, UNIROUEN, Inserm U1245, CHU Rouen, Department of Genetics and CNR-MAJ, FHU G4 Génomique, F-76000 Rouen, France; 72 Institute of Neurology, Catholic University of the Sacred Heart, School of Medicine, Milan, Italy; 73 Hospital de Cabueñes, Gijón, Spain; 74 Servicio de Neurología, Hospital Universitario de Gran Canaria Dr.Negrín, Las Palmas, Spain; 75 Inserm, Bordeaux Population Health Research Center, UMR 1219, Univ. Bordeaux, ISPED, CIC 1401-EC, Univ Bordeaux, Bordeaux, France; 76 CHU de Bordeaux, Pole de Santé Publique, Bordeaux, France; 77 Instituto de Medicina Molecular João lobo Antunes, Faculdade de Medicina, Universidade de Lisboa, Lisboa, Portugal; 78 Unit of Clinical Pharmacology, University Hospital of Cagliari, Cagliari, Italy; 79 Department of Psychology, University of California—Riverside, Riverside, CA USA; 80 University of Milan, Dino Ferrari Center, Milan, Italy; 81 VIB Center for Molecular Neurology, Antwerp, Belgium; 82 Laboratory of Neurogenetics, Institute Born-Bunge, Antwerp, Belgium; 83 Department of Biomedical Sciences, University of Antwerp., Antwerp, Belgium; 84 Insititute for Regenerative Medicine, University of Zürich, Zürich, Switzerland; 85 Glenn Biggs Institute for Alzheimer’s and Neurodegenerative Diseases, San Antonio, TX USA; 86 Department of Population Health Sciences, UT Health San Antonio, San Antonio, TX USA; 87 Division of Clinical Neurosciences, School of Medicine, University of Southampton, Southampton, UK; 88 Unidad de Trastornos del Movimiento, Servicio de Neurología y Neurofisiología, Instituto de Biomedicina de Sevilla (IBiS), Hospital Universitario Virgen del Rocío/CSIC/Universidad de Sevilla, Sevilla, Spain; 89 Department of Old Age Psychiatry, Institute of Psychiatry, Psychology & Neuroscience, King’s College London, London, UK; 90 Centre of Age-Related Medicine, Stavanger University Hospital, Stavanger, Norway; 91 Fondazione IRCCS Ca’ Granda, Ospedale Policlinico, Milan, Italy; 92 Normandie Univ, UNIROUEN, Inserm U1245, CHU Rouen, Department of Neurology and CNR-MAJ, FHU G4 Génomique, F-76000 Rouen, France; 93 Complex Structure of Geriatrics, Department of Medical Sciences Fondazione IRCCS Casa Sollievo della Sofferenza, San Giovanni Rotondo (FG), Italy; 94 Department of Child and Adolescent Psychiatry and Psychotherapy, Psychiatric University Hospital Zurich (PUK), University of Zurich, Zurich, Switzerland; 95 Neuroscience Center Zurich, University of Zurich and ETH Zurich, Zurich, Switzerland; 96 Zurich Center for Integrative Human Physiology, University of Zurich, Zurich, Switzerland; 97 School of Medicine, University of Thessaly, Larissa, Greece; 98 German Center for Neurodegenerative Diseases (DZNE), Magdeburg, Germany; 99 Institute of Cognitive Neurology and Dementia Research (IKND), Otto-von-Guericke University, Magdeburg, Germany; 100 School of Medicine and Surgery, University of Milano-Bicocca and Milan Center for Neuroscience, Milan, Italy; 101 Department of Neuroscience and Mental Health, AOU Città della Salute e della Scienza di Torino, Torino, Italy; 102 Neurological Tissue Bank of the Biobanc-Hospital Clinic-IDIBAPS, Institut d’Investigacions Biomèdiques August Pi i Sunyer, Barcelona, Spain; 103 Division of Neuropathology and Neurochemistry, Department of Neurology, Medical University of Vienna, Vienna, Austria; 104 APHP, Hôpital Brousse, equipe INSERM 1178, MOODS, Villejuif, France; 105 Université Paris-Saclay, UVSQ, Inserm, CESP, Team MOODS, Le Kremlin-Bicêtre, Paris, France; 106 APHP, Hôpital Broca, Paris, France; 107 School of Public Health, Human Genetics Center, University of Texas Health Science Center at Houston, Houston, TX USA; 108 Human Genome Sequencing Center, Baylor College of Medicine, Houston, TX USA; 109 Fondazione IRCCS Istituto Neurologico Carlo Besta, Milan, Italy; 110 Inserm U1172, CHU, DISTAlz, LiCEND, Univ Lille, Lille, France; 111 CHU CNR-MAJ, Lille, France; 112 Servicio de Inmunología, Hospital Universitario de Gran Canaria Dr. Negrín, Las Palmas de Gran Canaria, Spain; 113 Division of Clinical Geriatrics, Center for Alzheimer Research, Department of Neurobiology, Care Sciences and Society (NVS), Karolinska Institutet, Stockholm, Sweden; 114 Department of Psychiatry and Psychotherapy, University of Cologne, Medical Faculty, Cologne, Germany; 115 Excellence Cluster on Cellular Stress Responses in Aging-Associated Diseases (CECAD), University of Cologne, Cologne, Germany; 116 Norwegian National Advisory Unit on Ageing and Health, Vestfold Hospital Trust, Tønsberg, Norway; 117 Department of Geriatric Medicine, Oslo University Hospital, Oslo, Norway; 118 Institute of Clinical Medicine, University of Oslo, Oslo, Norway; 119 Department of Neurology, Medical School, University of Cyprus, Nicosia, Cyprus; 120 Laboratory of Neuropsychiatry, IRCCS Santa Lucia Foundation, Rome, Italy; 121 Beth K. and Stuart C. Yudofsky Division of Neuropsychiatry, Department of Psychiatry and Behavioral Sciences, Baylor College of Medicine, Houston, TX USA; 122 Aging Research Center, Department of Neurobiology, Care Sciences and Society, Karolinska Institutet and Stockholm University, Stockholm, Sweden; 123 MAC—Memory Clinic, IRCCS Istituto Centro San Giovanni di Dio Fatebenefratelli, Brescia, Italy; 124 Molecular Markers Laboratory, IRCCS Istituto Centro San Giovanni di Dio Fatebenefratelli, Brescia, Italy; 125 Sorbonne University, GRC n° 21, Alzheimer Precision Medicine (APM), AP-HP, Pitié-Salpêtrière Hospital, Paris, France; 126 EA 4468, Sorbonne Paris Cité, Université Paris Descartes, Paris, France; 127 Clinical Neurochemistry Laboratory, Sahlgrenska University Hospital, Mölndal, Sweden; 128 Department of Psychiatry and Neurochemistry, Institute of Neuroscience and Physiology, Sahlgrenska Academy at the University of Gothenburg, Gothenburg, Sweden; 129 Department of Neurodegenerative Disease, UCL Institute of Neurology, London, UK; 130 UK Dementia Research Institute at UCL, London, UK; 131 Institute of Clinical Medicine Neurology, University of Eastern Finland, Kuopio, Finland; 132 Neurocenter, neurology, Kuopio University Hospital, Kuopio, Finland; 133 Institute for Gerontology and Aging Research Network—Jönköping (ARN-J), School of Health and Welfare, Jönköping University, Jönköping, Sweden; 134 Neurology Unit, ‘San Gerardo’ hospital, Monza, Italy; 135 Department of Geriatrics, Clinic of Medicine, St Olavs Hospital, University Hospital of Trondheim, Trondheim, Norway; 136 Department of Neuromedicine and Movement Science, Norwegian University of Science and Technhology (NTNU), Trondheim, Norway; 137 Department of Neuroscience “Rita Levi Montalcini”, University of Torino, Torino, Italy; 138 Laboratorio de Genética, Hospital Universitario Central de Asturias, Oviedo, Spain; 139 Memory Clinic, Department of Neurology, 2nd Faculty of Medicine and Motol University Hospital, Charles University, Prague, Czech Republic; 140 International Clinical Research Center, St. Anne’s University Hospital Brno, Brno, Czech Republic; 141 Department of Psychiatry and Psychotherapy, School of Medicine Klinikum rechts der Isar, Technical University of Munich, Munich, Germany; 142 Population Health Unit, Finnish Institute for Health and Welfare, Helsinki, Finland; 143 Department of Psychiatry and Psychotherapy, University Medical Center Goettingen, Goettingen, Germany; 144 German Center for Neurodegenerative Diseases (DZNE), Goettingen, Germany; 145 Neurosciences and Signaling Group, Institute of Biomedicine (iBiMED), Department of Medical Sciences, University of Aveiro, Aveiro, Portugal; 146 Department of Psychiatry and Psychotherapy, Universitätsklinikum Erlangen, Friedrich-Alexander Universität Erlangen-Nürnberg, Erlangen, Germany; 147 Department of Population & Quantitative Health Sciences, Case Western Reserve University, Cleveland, OH USA; 148 Cleveland Institute for Computational Biology, Case Western Reserve University, Cleveland, OH USA; 149 Translational Neuroscience Laboratory, McLean Hospital, Harvard Medical School, Belmont, MA USA; 150 Department of Geriatric Psychiatry, University Hospital of Psychiatry Zürich, Zürich, Switzerland; 151 University of Zürich, Zürich, Switzerland; 152 Old age Psychiatry, University Hospital of Lausanne, Lausanne, Switzerland; 153 Department of Psychiatry, Psychosomatics and Psychotherapy, Center of Mental Health, University Hospital, Wuerzburg, Germany; 154 Institute for Stroke and Dementia Research, Klinikum der Universität München, Ludwig-Maximilians-Universität LMU, Munich, Germany; 155 German Center for Neurodegenerative Diseases (DZNE), Munich, Germany; 156 Schools of Life Sciences and Medicine, University of Nottingham, Nottingham, UK; 157 Department of Public Health and Caring Sciences/Geriatrics, Uppsala, Sweden; 158 Institute of Medical Biometry, Informatics and Epidemiology, University Hospital of Bonn, Bonn, Germany; 159 Departments of Medicine (Biomedical Genetics), Neurology, Ophthalmology, Epidemiology, and Biostatistics, Boston University Schools of Medicine and Public Health, Boston, MA USA; 160 Centre for Memory Disturbances, Lab of Clinical Neurochemistry, Section of Neurology, University of Perugia, Perugia, Italy; 161 Department of Geriatric Psychiatry, Central Institute for Mental Health Mannheim, Medical Faculty Mannheim, University of Heidelberg, Heidelberg, Germany; 162 Department of Epidemiology, Erasmus Medical Center, Rotterdam, The Netherlands; 163 1st Department of Neurology Aristotle University of Thessaloniki, Thessaloniki, Greece; 164 Azienda Ospedaliero-Universitaria, Parma, Italy; 165 Nuffield Department of Clinical Neurosciences, Oxford, UK; 166 Institute of Human Genetics, University of Bonn, School of Medicine & University Hospital Bonn, Bonn, Germany; 167 Munich Cluster for Systems Neurology (SyNergy), Munich, Germany; 168 Department of Primary Medical Care, University Medical Centre Hamburg-Eppendorf, Hamburg, Germany; 169 Laboratory of Cognitive Neuroscience, School of Psychology, Aristotle University of Thessaloniki, Thessaloniki, Greece; 170 Department of Nutrition and Dietetics, Harokopio University, Athens, Greece; 171 Institute of Gerontology and Geriatrics, Department of Medicine, University of Perugia, Perugia, Italy; 172 Institute of Public Health and Clinical Nutrition, University of Eastern Finland, Kuopio, Finland; 173 Neuroepidemiology and Ageing Research Unit, School of Public Health, Imperial College London, London, UK; 174 Stockholms Sjukhem, Research & Development Unit, Stockholm, Sweden; 175 Institute of Biomedicine, University of Eastern Finland, Kuopio, Finland; 176 Brown Foundation Institute of Molecular Medicine, University of Texas Health Sciences Center at Houston, Houston, TX USA; 177 1st Department of Neurology, Aiginition Hospital, National and Kapodistrian University of Athens, Medical School, Athens, Greece; 178 Taub Institute for Research in Alzheimer’s Disease and the Aging Brain, The Gertrude H. Sergievsky Center, Depatment of Neurology, Columbia University, New York, NY USA; 179 LVR-Hospital Essen, Department of Psychiatry and Psychotherapy, Medical Faculty, University of Duisburg-Essen, Essen, Germany; 180 Department of Psychiatry and Psychotherapy and Experimental and Clinical Research Center (ECRC), Charité-Universitätsmedizin Berlin, Berlin, Germany; 181 German Center for Neurodegenerative Diseases (DZNE), Berlin, Germany; 182 Bristol Medical School (THS), University of Bristol, Southmead Hospital, Bristol, UK; 183 Experimental Neuro-psychobiology Laboratory, Department of Clinical and Behavioral Neurology, IRCCS Santa Lucia Foundation, Rome, Italy; 184 Unit of Neuroscience, DIMEC, University of Parma, Parma, Italy; 185 Athens Association of Alzheimer’s disease and Related Disorders, Athens, Greece; 186 Institute of Medical Genetics and Pathology, University Hospital Basel, Basel, Switzerland; 187 Department of Psychiatry, Social Medicine Center East- Donauspital, Vienna, Austria; 188 Center of Mental Health, Clinic and Policlinic of Psychiatry, Psychosomatics and Psychotherapy, University Hospital of Würzburg, Würzburg, Germany; 189 Translational and Clincial Research Institute, Newcastle University, Newcastle upon Tyne, UK; 190 Campus for Ageing anf Vitality, Newcastle upon Tyne, UK; 191 Taub Institute on Alzheimer’s Disease and the Aging Brain, Department of Neurology, Columbia University, New York, NY USA; 192 Gertrude H. Sergievsky Center, Columbia University, New York, NY USA; 193 Department of Neurology, Columbia University, New York, NY USA; 194 Laboratory for Cognitive Neurology, Department of Neurosciences, University of Leuven, Leuven, Belgium; 195 Neurology Department, University Hospitals Leuven, Leuven, Belgium; 196 Center for Neurosciences, Vrije Universiteit Brussel (VUB), Brussels, Belgium; 197 Reference Center for Biological Markers of Dementia (BIODEM), University of Antwerp, Antwerp, Belgium; 198 Institute Born-Bunge, University of Antwerp, Antwerp, Belgium; 199 Department of Neurology, VUB University Hospital Brussels (UZ Brussel), Brussels, Belgium; 200 Institute of Clinical Medicine, Internal Medicine, University of Eastern Finland, Kuopio, Finland; 201 Department of Neurology and Clinical Neurophysiology, University Hospital of Trondheim, Trondheim, Norway; 202 Department of Neuromedicine and Movement Science, Faculty of Medicine and Health Sciences, Norwegian University of Science and Technology, Trondheim, Norway; 203 Department of Laboratory Diagnostics, III Laboratory of Analysis, Brescia Hospital, Brescia, Italy; 204 Alzheimer Research Center & Memory Clinic, Andalusian Institute for Neuroscience, Málaga, Spain; 205 MRC Prion Unit at UCL, Institute of Prion Diseases, London, UK; 206 Department of Medical Genetics, Oslo University Hospital, Oslo, Norway; 207 NORMENT, Department of Clinical Science, University of Bergen, Bergen, Norway; 208 Institute of Social Medicine, Occupational Health and Public Health, University of Leipzig, Leipzig, Germany; 209 Department of Neurology/CIEN Foundation/Queen Sofia Foundation Alzheimer Center, Madrid, Spain; 210 Azienda Ospedaliero-Universitaria Careggi Largo Brambilla, Florence, Italy; 211 UKDRI Cardiff, Cardiff University, Cardiff, UK; 212 Unitat de Genètica Molecular, Institut de Biomedicina de València-CSIC, Valencia, Spain; 213 Unidad Mixta de Neurologia Genètica, Instituto de Investigación Sanitaria La Fe, Valencia, Spain; 214 Department of Neurosciences, Faculty of Medicine and Nursery, University of the Basque Country, San Sebastián, Spain; 215 Centro de Biología Molecular Severo Ochoa (UAM-CSIC), Madrid, Spain; 216 Instituto de Investigacion Sanitaria ‘Hospital la Paz’ (IdIPaz), Madrid, Spain; 217 Departamento de Especialidades Quirúrgicas, Bioquímica e Inmunología. Facultad de Medicina, Universidad de Málaga, Málaga, Spain; 218 Fundación Pública Galega de Medicina Xenómica-CIBERER-IDIS, Santiago de Compostela, Spain; 219 Framingham Heart Study, Framingham, MA USA; 220 Nuffield Department of Population Health, University of Oxford, Oxford, UK; 221 Centre for Healthy Brain Ageing (CHeBA), School of Psychiatry, Faculty of Medicine, University of New South Wales, Sydney, NSW Australia; 222 Neuroscience Research Australia, Sydney, NSW Australia; 223 Department of Sociology, VU University, Amsterdam, The Netherlands; 224 NORMENT Centre, Institute of Clinical Medicine, University of Oslo, Oslo, Norway; 225 Division of Mental Health and Addiction, Oslo University Hospital, Oslo, Norway; 226 Department of Psychiatry, Glenn Biggs Institute for Alzheimer’s and Neurodegenerative Diseases, San Antonio, TX USA; 227 University of Oxford (OPTIMA), Oxford, UK; 228 OPTIMA, Department of Pharmacology, University of Oxford, Oxford, UK; 229 Dirección de Atención de Adultos Mayores del Min., Salud Desarrollo Social y Deportes de la Pcia. de Mendoza, Mendoza, Argentina; 230 Geriatrics Unit Fondazione Policlinico A. Gemelli IRCCS, Rome, Italy; 231 Cerebral Function Unit, Greater Manchester Neurosciences Centre, Salford Royal Hospital, Salford, UK; 232 Division of Neuroscience and Experimental Psychology, School of Biological Sciences, Faculty of Biology, Medicine and Health, University of Manchester, Manchester, UK; 233 Centre National de Recherche en Génomique Humaine (CNRGH), Institut de Biologie François Jacob, CEA, Université Paris-Saclay, F-91057 Evry, France; 234 Department of Neurology, Kuopio University Hospital, Kuopio, Finland; 235 Insitute of Clinical Medicine-Neurology, University of Eastenr Finland, Kuopio, Finland; 236 Institute of Neurology, Catholic University of Sacred Heart, Fondazione Policlinico Universitario A. Gemelli IRCCS, Rome, Italy; 237 Research Laboratory, Complex Structure of Geriatrics, San Giovanni Rotondo, Fondazione IRCCS Casa Sollievo della Sofferenza, Foggia, Italy; 238 Centre for Public Health, School of Medicine, Queen’s University Belfast, Belfast, UK; 239 Clinica Medica “Frugoni” and Geriatric Medicine-Memory Unit, University of Bari Aldo Moro, Bari, Italy; 240 CENECON-FMED-UBA, Buenos Aires, Argentina; 241 ENERI, Vienna, Austria; 242 Htal Santojani, Buenos Aires, Argentina; 243 Servicio de Neurología, Hospital de Cabueñes, Gijón, Spain; 244 UBA, Buenos Aires, Argentina; 245 HIGA Eva Perón, Billinghurst, Argentina; 246 University of Oxford, Oxford, UK; 247 Neurología Clinica, Madrid, Spain; 248 German Center for Neurodegenerative Diseases (DZNE), Tübingen, Germany; 249 Section for Dementia Research, Hertie Institute for Clinical Brain Research and Department of Psychiatry and Psychotherapy, University of Tübingen, Tübingen, Germany; 250 Hospital Dr. Lucio Molas, Santa Rosa, Argentina; 251 Fundacion Ayuda Enfermo Renal y Alta Complejidad (FERNAC), Santa Rosa, Argentina; 252 CONICET, Buenos Aires, Argentina; 253 Institute for Stroke and Dementia Research (ISD), University Hospital, Ludwig-Maximilian University Munich, Munich, Germany; 254 Faculty of Medical and Human Sciences, Institute of Brain, Behaviour and Mental Health, Manchester, UK; 255 Clinic of Neurology UH ‘Alexandrovska’, Medical University—Sofia, Sofia, Bulgaria; 256 Fundacion Sinapsis, Santa Rosa, Argentina; 257 Laboratory of Brain Aging and Neurodegeneration- FIL, Buenos Aires, Argentina; 258 IIBBA (CONICET), Buenos Aires, Argentina; 259 Salford Royal NHS Foundation Trust, Salford, England; 260 Nuffield Department of Clinical Neurosciences, University of Oxford, Oxford, UK; 261 Department of Psychiatry, University of Bonn, Bonn, Germany; 262 Department of Radiology, University Hospital Bonn, Bonn, Germany; 263 Department of Public Health, University of Helsinki, Helsinki, Finland; 264 National School of Public Health, Madrid, Spain; 265 South Ostrobothnia Central Hospital, Seinäjoki, Finland; 266 Dementia Research Centre, Department of Neurodegenerative Disease, UCL Queen Square Institute of Neurology, University College London, London, UK; 267 Department of Neuropsychiatry, Charité, Berlin, Germany; 268 ENYS (Estudio en Neurociencias y Sistemas Complejos) CONICET-Hospital El Cruce ‘Nestor Kirchner’-UNAJ, Buenos Aires, Argentina; 269 Molecular Medicine Center, Department of Medical chemistry and biochemistry, Medical University of Sofia, Sofia, Bulgaria; 270 Laboratory of Neurochemistry, UZ Brussel, Brussel, Belgium; 271 Unit of Clinical Pharmacology, Teaching Hospital of Cagliari, AOUCA, Cagliari, Italy; 272 Programa del Adulto Mayor-Min, Salud de la Pcia. de Jujuy, San Salvador de Jujuy, Argentina; 273 Division of Neuroscience and Experimental Psychology, University of Manchester, Manchester, UK; 274 UK Dementia Research Institute at UCL, UCL Queen Square Institute of Neurology, University College London, London, UK; 275 Center for Life Course Health Research, University of Oulu, Oulu, Finland; 276 Medical Research Center Oulu, Oulu University Hospital, Oulu, Finland; 277 Oulu City Hospital, Oulu, Finland; 278 German Center for Neurodegenerative Diseases (DZNE, Munich), Munich, Germany; 279 Department of Psychiatry and Psychotherapy, University Hospital LMU Munich, Munich, Germany; 280 Ageing Epidemiology Research Unit (AGE), School of Public Health, Imperial College London, London, UK; 281 University of Bristol Institute of Clinical Neurosciences, School of Clinical Sciences, Frenchay Hospital, Bristol, UK; 282 German Center for Neurodegenerative Diseases (DZNE), Rostock, Germany; 283 Department of Psychosomatic Medicine, Rostock University Medical Center, Rostock, Germany; 284 Finnish Institute for Health and Welfare, Helsinki, Finland; 285 Joint Municipal Authority for North Karelia Social and Health Services (Siun Sote), Joensuu, Finland; 286 Helsinki University Hospital, University of Helsinki, Helsinki, Finland; 287 Department of Clinical Biochemistry, Hematology and Immunology, Na Homolce Hospital, Prague, Czech Republic; 288 Servei de Neurologia, Hospital Clínic Universitari de València, València, Spain; 289 Department of Neurology, Hospital Universitario Son Espases, Palma, Spain; 290 Hospital Universitario Ramón y Cajal, Madrid, Spain; 291 BIOMICs, País Vasco, Centro de Investigación Lascaray, Universidad del País Vasco UPV/EHU, Leioa, Spain; 292 Fundación para la Formación e Investigación Sanitarias de la Región de Murcia, El Palmar, Spain; 293 Fundación CITA-alzheimer, Centro de Investigacio´n y Terapias Avanzadas, San Sebastián, Spain; 294 Navarrabiomed, Pamplona, Spain; 295 Barcelona beta Brain Research Center–Fundació Pasqual Maragall, Barcelona, Spain; 296 Hospital Universitario La Princesa, Madrid, Spain; 297 School of Health Sciences, Bangor University, Bangor, UK; 298 Institute of Psychology, Health and Society, University of Liverpool, Liverpool, England; 299 School of Clinical Medicine, Cambridge Institute of Public Health, University of Cambridge, Cambridge, England; 300 Faculty of Medicine, Institute of Health and Society, University of Newcastle, Callaghan, NSW Australia; 301 Dementia Studies, University of Stirling, Stirling, Scotland; 302 School of Psychology, University of Liverpool, Liverpool, England; 303 School of Psychology, Centre for Research in Ageing and Cognitive Health (REACH), University of Exeter, Exeter, England; 304 Dementia Services Development Centre, Bangor University, Wales, UK; 305 Swansea University, School of Human Sciences, Centre for Innovative Ageing, Swansea, UK

**Keywords:** Genomics, Cognitive ageing, Risk factors

Correction to: *Nature Communications* 10.1038/s41467-021-22491-8, published online 07 June 2021

The original version of this Article omitted from the author list the 212th author Patrizia Mecocci, who is from the Institute of Gerontology and Geriatrics, Department of Medicine, University of Perugia, Perugia, Italy. Consequently, the “Sample Contribution” section of Author Contributions was updated to add “P.M” between “P.D.” and “R.C.”. Additionally, the original version of this Article contained the incorrect affiliation for author Patrick Gavin Kehoe, which incorrectly read “German Center for Neurodegenerative Diseases (DZNE), Berlin, Germany”. The correct version replaces this affiliation with “Bristol Medical School (THS), University of Bristol, Southmead Hospital, Bristol, UK”. This has been corrected in both the PDF and HTML versions of the Article.

