# Supplementary material for: Common variants in Alzheimer’s disease and risk stratification by polygenic risk scores
Source: Nat Commun. 2021 Jun 7;12:3417. doi: 10.1038/s41467-021-22491-8 (PMC8184987; doi:10.1038/s41467-021-22491-8)
Supplement: Supplementary file 1 — Supplementary Information [file 41467_2021_22491_MOESM1_ESM.pdf]

Common variants in Alzheimer's disease and stratification by polygenic risk scores

Supplementary Information

Supplementary Methods

Functional analysis of *APP*
DNA was extracted using standard procedures, and *APP* rs2154481 was analyzed using a specific Taqman® SNP genotyping assay (Life Technologies, Carlsbad, CA) in the 7900-HT Fast Real-Time PCR System (Applied Biosystems, Foster City, CA). Total RNA was isolated from 60 mg of frozen cerebellum specimens, obtained from the Neurological Tissue Bank (Barcelona). Brain tissue was homogenized in liquid nitrogen and transferred into the TRIzol reagent (Life Technologies, Carlsbad, CA). The conventional phenol–chloroform protocol was followed by a column-based RNA purification method using the RNeasy Mini Kit (Qiagen, Hilden, Germany). Reverse transcription was performed using the RevertAid First Strand cDNA Synthesis Kit (Thermo Scientific, Rockford, IL).

Expression analyses of APP lncRNA and mRNA were performed by a quantitative polymerase chain reaction (qPCR) using Fast SYBR Green Master Mix (Thermo Scientific, Rockford, IL) and 200 nM of each primer. The following primer sequences were used for APP lncRNA and mRNA: Forward 5'-AAGAGAAGAACCGTGTGCTAAC-3', Reverse 5'-GCTATTGAAGGCTGACATTGAGA-3'; and Forward 5'-CCATTTCCAGAAAGCCAAAG-3', Reverse 5'-TCTGGCCATGTGTGTCTCC-3', respectively. The samples were assayed in duplicate and normalized to GAPDH (Forward 5'-TGCACCACCAACTGCTTAGC-3'; Reverse 5'-GCCATGGACTGTGGTCATGAG-3').

The expression levels were determined by the 2-ΔΔCt method. The Kruskal–Wallis test with Dunn's post hoc analysis was applied for comparisons among diagnostic groups, and Spearman's rank coefficient was used for correlation among expression analyses. Adjusted statistical significance was set at $\alpha = 0.05$. Built-in functions and "ggplot2" (v3.1.1, Wickham 2016) implemented in R statistical software (v3.6.0) were used for plots and statistical analyses.

Neuropathological examination and disease evaluation were performed at the Neurological Tissue Bank of the Biobanc Hospital Clinic–Institut d'Investigacions Biomèdiques August Pi i Sunyer (IDIBAPS) according to standardized protocols and international consensus criteria. The study (IIBSP-DFT-2018–97) was approved by the local ethics committees of both the Neurological Tissue Bank and the Sant Pau Research Institute (Barcelona, Spain).

Functional annotation by FUMA[1]
Annotation of candidate SNPs in genomic risk loci
The functional effects of SNPs on genes were obtained by performing ANNOVAR[3] using Ensembl genes (build 85). Note that SNPs can be annotated to more than one gene in the case of intergenic SNPs, which are annotated to the two closest up- and downstream genes. CADD scores[4], RegulomeDB scores[5], and the 15-core chromatin state[6] are annotated to all SNPs in 1000 Genomes phase 3 by matching chromosome, position, reference and alternative alleles.

Positional mapping maps SNPs to genes based on physical distance (within a 10 Kb window) from known protein-coding genes in the human reference assembly (GRCh37/hg19). Two scores of intolerance to functional mutations are annotated: the probability of being loss-of-function intolerant[7] (pLI) and the noncoding residual variation intolerance score[8] (ncRVIS).

eQTL mapping maps significant SNPs and SNPs in LD to genes with which they show a significant eQTL association or where the same allelic variation at the SNP is associated with the expression level of that gene. eQTL mapping uses information from 45 tissue types in 3 data repositories (GTEx[9] v6, Blood eQTL browser[10], BIOS QTL browser[11]) and is based on cis-eQTLs, which can map SNPs to genes up to 1 Mb apart. We used a false discovery rate of 0.05 to define significant eQTL associations. Chromatin interaction mapping was performed to map SNPs to genes given three-dimensional DNA–DNA interaction between the SNP region and another gene region.

MAGMA for gene analysis and gene set analysis
FUMA uses input GWAS summary statistics to compute gene-based P-values (gene analysis) and gene set P-values (gene set analysis) using the MAGMA[12] tool. For gene analysis, the gene-based P-value is

computed for protein-coding genes by mapping SNPs to genes if the SNPs are located within the genes. For gene set analysis, the gene set P-value is computed using the gene-based P-value for 4,728 curated gene sets (including canonical pathways) and 6,166 GO terms obtained from MsigDB v5.2. For both analyses, the default MAGMA setting (the SNP-wise model for gene analysis and the competitive model for gene set analysis) was used, and the Bonferroni correction (gene) or FDR (gene set) was used to correct for multiple testing.

Gene expression dataset and gene set enrichment test
The GENE2FUNC process annotates the prioritized genes in a biological context and visualizes their tissue-specific expression patterns as an interactive heatmap based on GTEx v6 RNA-seq data for each gene. Normalized gene expression data (RPKM or read per kilobase per million) from the GTEx portal v68 for 53 tissue types was processed. To test for the overrepresentation of biological functions, a list of prioritized genes was tested against gene sets obtained from MsigDB and WikiPathways using hypergeometric tests. FUMA reported gene sets with an adjusted P-value of ≤ 0.05, and the number of genes that overlap with the gene set was > 1 by default. Multiple testing correction was performed per data source of the tested gene sets.

Expression data in public databases
We use GTEx Portal[9] and BrainSeq[13] eQTL Browser to identify expression quantitative trait loci (eQTLs) in the genome wide significant loci in this study. For GTEx we download the multi-tissue eQTL comparison (data Source: GTEx Analysis Release V8 (dbGaP Accession phs000424.v8.p2), see details in https://gtexportal.org/home/.
The BrainSeq is based on the dorsolateral prefrontal cortex (DLPFC) polyA+ RNA-seq on 738 subjects spanning the lifespan and three main psychiatric diagnostic groups (Schizophrenia, Major Depression Disorder, and Bipolar Disorder). BrainSeq identified eQTLs in the DLPFC using RNA sequencing (RNA-seq) and genotype data. The eQTL modeling tested for additive genetic effects on expression while adjusting for sex, ancestry (multidimensional scaling components), and expression heterogeneity (principal components). Significant eQTLs were those SNP-feature pairs with a false discovery rate (FDR) less than 1%. The "DLPFC - All" database was used. For more details, see http://eqtl.brainseq.org/phase1/eqtl/.

Follow-up datasets
We present short descriptions of the 16 cohorts, often including multiple sites or studies that contributed to this manuscript (Supplementary Data 1).

The European Alzheimer's Disease DNA Biobank dataset (EADB) contains 14,546 AD cases and 19,207 controls analyzed mainly from four nodes: EADB-France, EADB-Germany, EADB-Netherlands, and EADB-Australia.

EADB-France
In the France node (*EADB-nodeFRA*), samples were collected from nine countries (39 centers), and after processing the data, we determined 10,374 AD cases and 11,321 controls (Supplementary Data 12): Belgium (552 AD cases and 1,040 controls), the Czech Republic (341 AD cases and 35 controls), Finland (668 AD cases and 1,235 controls), France (2,166 AD cases and 3,749 controls), Greece (536 AD cases and 103 controls), Italy (2,636 AD cases and 1,080 controls), Spain (470 AD cases and 533 controls), Sweden (755 AD cases and 1,454 controls), and the United Kingdom (2,230 AD cases and 2,092 controls).

Belgium: The participants were part of a large prospective cohort[14] of Belgian AD patients and healthy elderly control individuals. The patients were ascertained at the memory clinic of Middelheim and Hoge Beuken (Hospital Network Antwerp, Belgium) and at the memory clinic of the University Hospitals of Leuven, Belgium. The control individuals were the partners of the patients or volunteers from the Belgian community. The study protocols were approved by the ethics committees of the Antwerp University Hospital and the participating neurological centers at the different hospitals of the BELNEU consortium and by the University of Antwerp.

Czech Republic: The Czech Brain Aging Study[15] (CBAS) is a longitudinal memory-clinic–based study recruiting subjects at risk of dementia (subjects referred for cognitive complaints—SCD, MCI). The CBAS+ study is a cross-sectional study of patients in the early stages of dementia. Both studies were signed by all subjects and were approved by the local ethics committee.

Finland: The ADGEN cohort[16] is a clinic-based collection of AD patients from Eastern and Northern Finland examined in the Department of Neurology in Kuopio University Hospital and the Department of Neurology in Oulu University Hospital. All the patients were diagnosed with probable AD according to the criteria of the National Institute of Neurological and Communicative Disorders and Stroke and the Alzheimer's disease and Related Disorders Association (NINCDS–ADRDA). The study was approved by the ethics committee of Kuopion University Hospital, Finland (420/2016).

The FINGER study[17] is a Finnish multi-domain lifestyle RCT enrolling 1,260 older adults with an increased risk of dementia from the general population. The intensive lifestyle intervention lasted for two years, and follow-up extends currently up to seven years. The FINGER study was approved by the coordinating ethics committee of the Hospital District of Helsinki and Uusimaa (94/13/03/00/2009 and HUS/1204/2017), and all the participants gave written informed consent.

France: The BALTAZAR multicenter (23 memory centers) prospective study[18] included 1,040 participants from September 2010 to April 2015. They were classified as AD cases (n = 501) according to DSM IV-TR and NINCDS–ADRDA criteria as well as amnestic mild cognitive impairment (MCI) cases (a MCI, n = 417) and non-amnestic MCI cases (na MCI, n = 122) according to Petersen's criteria. A comprehensive battery of cognitive tests was performed, including MMSE, verbal fluency, and FCSRT. All the participants or their legal guardians gave written informed consent. The study was approved by the Paris ethics committee (CPP Ile de France IV Saint Louis Hospital).

MEMENTO is a clinic-based study[19] aimed at better understanding the natural history of AD, dementia, and related diseases. Between 2011 and 2014, 2,323 individuals presenting either recently diagnosed MCI or isolated cognitive complaints were enrolled in 26 memory centers in France. This study was performed in accordance with the guidelines of the Declaration of Helsinki. The MEMENTO study protocol has been approved by the local ethics committee (Comité de Protection des Personnes Sud-Ouest et Outre Mer III; approval number 2010-A01394-35). All the participants provided written informed consent.

The CNRMAJ-Rouen study[20] provided early onset AD patients (n = 870). The patients or their legal guardians provided written informed consent. This study was approved by the ethics committee of CPP Ile de France II. The Karolinska Institutet AD cohort participated with AD cases (n = 444). All parts were approved by the ethics committee (Dnr484/02 och 485/02 and 2017/834-31/1).

Italy: The Milan samples were progressively recruited during different study[21–23] protocols. The cohort is composed of outpatients with AD referred to the geriatric unit of the Fondazione IRCCS Ca' Granda Ospedale Maggiore Policlinico (Milan, Italy). The control group is constituted by age-matched octogenarians without cognitive decline. An ethical statement was attained from the Fondazione IRCCS Ca' Granda Ospedale Maggiore Policlinico. All the subjects gave their informed consent to participate in the study. The PoliMi cohort was formed by consecutive patients referred to the Alzheimer Center of the Policlinico Academic Hospital (Milan, Italy).

Spain: The participants of the Sant Pau Initiative on Neurodegeneration[24] (SPIN) cohort provided informed consent to donate blood and cerebrospinal fluid samples, receive detailed neurological and neuropsychological evaluations, and undergo structural 3T brain MRI scans. The participants are followed annually for a minimum of four years, with repeated cerebrospinal fluid collection and imaging studies performed every other year, and brain donation is encouraged. At the moment of inclusion, the details of the protocol are explained, and verbal and signed informed consent is obtained from all the participants. All the protocols were approved by the ethics committee of the Sant Pau Hospital.

Sweden: Swedish National Study on Aging and Care in Kungsholmen (SNAC-K) data was collected. The original SNAC-K population consisted of 4590 living and eligible persons who lived on the island of Kungsholmen in Central Stockholm, belonged to pre-specified age strata, and were randomly selected to take part in the study. Between 2001 and 2004, 3363 persons participated in the baseline assessment. They belonged to the age cohorts 60, 66, 72, 78, 81, 84, 87, 90, 93, and 96 years and 99 years and older.

The examination consists of three parts: a nurse interview, a medical examination, and a neuropsychological testing session. Altogether, the examination takes about six hours. The participants are reexamined each time they reach the next age cohort. All parts of the SNAC-K project have been approved by the ethical committee at Karolinska Institutet or the regional ethical review board. Informed consent was collected from all the participants or, if the person was severely cognitively impaired, from their next of kin.

EADB-German

In the German node, samples were collected from seven countries: Germany, Greece, Switzerland, Portugal, Spain, Belgium, and Bulgaria. To avoid problems of population stratification, each country with AD and controls was analyzed separately.

EADB-GER was configured by seven datasets from Germany comprising 1,228 AD cases and 1,799 controls: the VOGEL study, the Heidelberg/Mannheim memory clinic sample, the PAGES study, the Technische Universität München study, the Göttingen Universität study, the German Dementia Competence Network (DCN) cohort, and the German Study on Aging, Cognition, and Dementia (AgeCoDe).

The VOGEL study: The VOGEL study is a prospective, observational, long-term follow-up study with three time points of investigation within 6–8 years. This cohort includes dementia and healthy subjects. Residents of the city of Würzburg born between 1936 and 1941 were recruited. Every participant underwent physical, psychiatric, and laboratory examinations and performed intense neuropsychological testing as well as VSEP and NIRS according to the published procedures. A total of 604 subjects were included. The mean age of the participants was 73.9 ± 1.55 years.

The Heidelberg/Mannheim memory clinic sample: This cohort includes 61 subjects from whom 40 MCI patients were recruited and assessed between 2012 and 2016. Some of those patients converted to dementia by AD or other dementias.

The PAGES study: This study includes 301 subjects. AD patients were recruited at the memory clinic of the Department of Psychiatry, University of Munich, Germany. Participants in whom dementia associated with AD was diagnosed fulfilled the criteria for probable AD according to the NINCDS–ADRDA. The control group included participants who were randomly selected from the general population of Munich. Controls who had central nervous system diseases or psychotic disorders or who had first-degree relatives with psychotic disorders were excluded.

The Technische Universität München study: This cohort includes 359 healthy, AD, and other dementias patients recruited from the Centre for Cognitive Disorders. All the participants provided written informed consent. A biobank was submitted to the ethics committee of the Technical University of Munich, School of Medicine (Munich, Germany), which raised no objections and approved the biobank (reference number 347-14).

The Göttingen Universität study: This study includes 111 in- and outpatients with a healthy or AD dementia status from the Department of Psychiatry of the University of Göttingen. The study's ethical statement was provided locally at the Göttingen University Medical Centre.

The German Dementia Competence Network (DCN) cohort: Individuals from the DCN cohort were recruited from 14 university hospital memory clinics across Germany between 2003 and 2005[25]. The study was approved by the respective ethics committees, and written informed consent was obtained from all the participants prior to inclusion.

The German Study on Aging, Cognition, and Dementia (AgeCoDe): The AgeCoDe study is a general practice (GP) registry-based longitudinal study in elderly individuals that recruited patients aged 75 years and above in six German cities from 2003 to 2004.[26] The study was approved by the respective ethics committees, and written informed consent was obtained from all the participants prior to inclusion.

EADB-SWI was configured by two datasets from Switzerland and Austria, totaling 181 AD cases and 401 controls: the Lausanne study and the VITA study.

The Lausanne study: This study includes 137 community-dwelling participants aged 55+ years with cognitive impairment (memory clinic patients with MCI, dementia) or normal cognition (recruited by advertisement, word of mouth). The study's ethical statement was provided locally at the Department of Psychiatry, Geneva University Centre, Switzerland.

The VITA study: This is a longitudinal study of 606 individuals (Vienna, Austria) who were 75 years old in 2000, followed up every 30–90 months. This cohort includes dementia and healthy subjects. All the participants gave written informed consent. The study conformed to the latest version of the Declaration of Helsinki and was approved by the ethics committee of the City of Vienna, Austria.

EADB-GRE was configured by the HELIAD study from Greece, comprising 49 AD cases and 1,150 controls. HELIAD is a population-based, multidisciplinary, collaborative study designed to estimate, in the Greek population over the age of 64 years, the prevalence and incidence of MCI, AD, other forms of dementia, and other neuropsychiatric conditions of aging and to investigate associations between nutrition and cognitive dysfunction or age-related neuropsychiatric diseases. The participants were selected through random sampling from the records of two Greek municipalities, Larissa and Marousi. All the participants signed informed consent in Greek.

EADB-POR was configured by the Lisbon study from Portugal, totaling 78 AD cases and 74 controls. This cohort was recruited in 2008–2009 to investigate the connections between oxidative stress and lipid dyshomeostasis in AD. The project includes 190 subjects and was approved by the local ethics committee, and all the participants provided written informed consent. This study includes healthy and dementia-by-AD subjects.

EADB-F.ACE/BBB represents the Spanish samples. This sub-cohort configured an extra and independent dataset comprising 1008 AD cases and 1386 controls. A fraction of the AD cases (n = 332) were pathological, confirmed by the Biobanc Hospital Clínic–IDIBAPS[27] (Barcelona, Spain; www.clinicbiobanc.org), following the guidelines and approval of the local ethics committee. The remaining cases (n = 676) were diagnosed based on clinical criteria from Fundació ACE. These individuals were recruited following a methodology identical to that previously described for the GR@ACE discovery cohort.

EADB-Netherlands

The *EADB–NL* dataset contains 1,895 AD cases and 2,865 controls recruited by three studies: the Amsterdam Dementia Cohort (ADC), the Longitudinal Aging Study of Amsterdam (LASA), and the 100-Plus study. The Medical Ethics Committee (METC) of the VU University Medical Centre approved the three studies. All the participants and/or their legal guardians gave written informed consent for participation in the clinical and genetic studies.

The 100-Plus study: This study includes Dutch-speaking individuals who (i) can provide official evidence for being aged 100 years or older, (ii) self-report to be cognitively healthy, which is confirmed by a proxy, (iii) consent to the donation of a blood sample, (iv) consent to (at least) two home visits from a researcher, and (v) consent to undergo an interview and neuropsychological test battery[28].

The Longitudinal Aging Study of Amsterdam (LASA): This ongoing longitudinal study, initiated in 1991, represents a sample of individuals aged 55–85 years from the Netherlands[29,30].

The Amsterdam Dementia Cohort (ADC): This cohort comprises patients who visit the memory clinic of the VU University Medical Centre, the Netherlands. The diagnosis of probable AD is based on the clinical criteria formulated by the NINCDS–ADRDA and based on the NIA–AA[31,32].

EADB-Australia

The Sydney MAS study is a longitudinal study investigating MCI, related syndromes, and age-related cognitive change. Older adults (70–90 years old) were randomly recruited from the community in Sydney, Australia (n = 1,037). An extensive interview was undertaken and questionnaire data collected, including demographics, cognitive performance, and medical history. The majority of participants provided blood samples for genetic analysis. Neuroimaging was performed on a subset of participants. Ethics approval for

the study was provided by the ethics committee of the University of New South Wales and the Illawarra Area Health Service Human Research Ethics Committee. All the participants provided written informed consent to join the study. More information is provided in Sachdev et al.[33] In our study, we included 43 cases and 215 controls.

The Alzheimer workgroup initiative of the Psychiatric Genomic Consortium (PGC–ALZ) was configured by three nonpublic datasets with 2,737 AD cases and 14,800 controls (the Norwegian DemGene network, the Swedish Twin Studies of Aging, and TwinGene). Further details can be found in Jansen et al.[2].

> The Norwegian DemGene Network: This is a Norwegian network of clinical sites collecting cases from memory clinics based on a standardized examination of cognitive, functional, and behavioral measures and data on the progression of most patients. The Norwegian DemGene Network includes 2,224 cases and 1,855 healthy controls from different studies described elsewhere[2]. The cases were diagnosed according to recommendations from the NIA–AA, the NINCDS–ADRDA criteria, or the ICD-10 research criteria. The controls were screened with a standardized interview and cognitive tests. Individuals from the DemGene study were genotyped using the Human Omni Express-24 v1.1 (Illumina Inc., San Diego, CA) at deCODE Genetics (Reykjavik, Iceland).
>
> The Swedish Twin Studies of Aging (STSA): These include three sub-studies of aging within the Swedish Twin Registry (n cases = 398, n controls = 1,079). Informed consent was obtained from all the participants, and the studies were approved by the regional ethics board in Stockholm and the institutional review board at the University of Southern California. DNA was extracted from blood samples and genotyped using Illumina Infinium PsychArray. The AD patients were diagnosed as part of the studies according to the NINCDS–ADRDA criteria.
>
> TwinGene: This is a population-based study of older twins drawn from the Swedish Twin Registry. Written informed consent was obtained from all the participants, and the study was approved by the regional ethics board in Stockholm. DNA was extracted from blood samples and genotyped using Illumina Human OmniExpress.

We also configured the *GR@ACE replication*, an extra and independent dataset comprising 516 AD cases and 686 controls. This dataset was incorporated only for replication purposes. The GR@ACE dataset was recruited following a methodology identical to that described for the GR@ACE discovery cohort.

The Clinical AD Sweden and the Gothenburg Birth Cohort Studies (GBCS) consist of AD cases recruited at memory clinics in different Swedish cities[34]. The study was approved by the local ethical commissions at the respective academic centers, and the tenets of the Declaration of Helsinki were followed. In our study, the AD and GBCS cohorts contributed to 514 AD cases and 3,467 controls. The GBCS cohort consists of data from four studies that recruited individuals representative of the Swedish population (controls). The sample with genetic data includes individuals from the Prospective Population Study of Women (PPSW), on women who have been followed since 1968 (age at inclusion 38–60)[35]; individuals born in 1930 and 1944 from the H70 study, which includes longitudinally followed samples recruited at age 70[36,37]; individuals born in 1923–1924 from the H85 study, which includes longitudinally followed samples recruited at age 85[38]; and individuals from the 95-Plus study, recruited between 1996 and 2012 and followed until death[39]. The studies were approved by the regional ethical review board in Gothenburg, and informed consent was obtained from all the participants and/or their relatives in cases of dementia.

The Neocodex–Murcia study (NxC) includes 324 sporadic AD patients and 754 controls of unknown cognitive status from the Spanish general population collected by Neocodex[40,41]. AD patients were diagnosed as having possible or probable AD in accordance with the NINCDS–ADRDA criteria[42].

The AddNeuroMed Study (ADDN) was a public–private partnership for biomarker discovery and replication in AD[43–45]. It was a multicenter study in Europe, with the first patient enrolled in January 2006 and the last in February 2008. The study protocol was planned for a baseline assessment visit, with follow-ups every three months for the first year and then annual visits that continued through 2013. The study enrolled a total of 258 AD, 257 MCI, and 266 controls, but not all had complete data at each assessment. In our study, we included 450 cases and 187 controls. This dataset was downloaded from Synapse[46].

Supplementary Figures

Supplementary Figure 1. **Genome-wide association study. a-c,** Principal component analysis and QQ-plot for the GR@ACE dataset. **d,** QQplot Discovery meta-analysis. **e,** Correlation between the effect estimates from the AD case-control and AD by proxy approach for the significant loci. We compared the results obtained to a second meta-analysis using only the case-control datasets (IGAP Stages 1–2) and GR@ACE datasets as a sensitivity analysis to identify false negative results given possible dilution by the by-proxy approach in the UK Biobank (Supplementary Data 3). The meta-analysis, including the by-proxy summary statistics, identified 11 additional loci reaching genome-wide significance with respect to case-control-only results. The incorporation of by-proxy summary statistics did not show an association in two previously reported AD loci (rs7185636-*IQCK* and rs386572859-*MAPT*) by the IGAP consortium and replicated in the GR@ACE dataset (OR = 0.93[0.90–0.95], p = 4.5×10^{-8} and OR = 0.81[0.75–0.87], p = 7.9×10^{-9}, respectively).....................................9

Supplementary Figure 2. **Forest plots for the six novel signals identified in overall meta-analysis**. See sample size in Supplementary Data 1. Data are presented as Odds Ratio (95% CI). *10*

Supplementary Figure 3. **LocusZoom and forest plots: strengthened evidence of association with AD for three additional genomic loci. a,** *HS3ST1* loci. **b,** *IL34* loci and **c,** *PLCG2* loci. See sample size in Supplementary Data 1. Data for the forest plots are presented as Odds Ratio (95% CI). The first was rs4351014 with AD (combined OR = 0.94 [0.92–0.95], p = 9.2×10^{-12}). This variant is in a gene-poor region but has been previously linked to *HS3ST1*. The second was a stop codon mutation (rs4985556, Tyr213Ter, MAF = 0.111) in the interleukin 34 (*IL34*) gene that was previously reported (Marioni et al. 2018) to be associated in a by-proxy approach (combined OR = 1.08 [1.06–1.11], p = 3.9×10^{-10}). The third genomic region contains the *PLCG2* gene, which has been associated with AD twice before (the rare missense variant p.P522R in the *PLCG2* gene and rs12444183 near the promotor region of *PLCG2*). After the combination of discovery and follow-up, a third independent association signal emerged in the *PLCG2* region (rs3935877, effect allele frequency = 0.868, OR = 0.92 [0.90–0.95], p = 6.9×10^{-9}). We also strengthened the association of *PLCG2*-rs12444183 with AD (MAF = 0.407, combined OR = 0.95 [0.93–0.96], p = 6.8×10^{-12}). Conditional analyses in the *PLCG2* region showed that the association signals of all three variants (including the missense variant p.P522R in *PLCG2*) are independent (Supplementary Data 13). A conditional analysis on the nearby *SCIMP* locus (333 Kb) (Supplementary Data 14) showed similar effects after adjustment for *SCIMP* (and vice versa), in line with the fact that the two independent signals are in weak LD (R2 = 0.139, D' = 0.446). *11*

Supplementary Figure 4. **Functional analysis. a,** Diagram of functional interpretation by 4 FUMA strategies. To link the novel variants to specific genes and functional motifs in their genomic regions, we applied different strategies implemented on the FUMA platform. FUMA helps to generate hypotheses that are testable in functional experiments aimed at proving causal relations. The genes *APP, IL34, CHRNE, PLCG2,* and *SHARPIN* were the most likely candidate genes in the regions as they were implicated in at least three mapping strategies (Supplementary Data 15–18). **b,** Differential tissue expression for APP eQTL according to GTEx. Data are presented as Normalized effect size (NES) with 95% CI. **c,** Differential expression of lncAPP and mRNA in AD cases and controls (n=34 lncAPP and n=31 mRNA biologically independent samples). No significant differences were found between the total expression of the lncAPP (AP001439.2) and

mRNA expression in the brain case/control samples. **d**, Differential expression of lncAPP and mRNA stratified by genotype. The allelic frequency was as expected (MAF = 0.41), as well as the eQTL effect for mRNA (CC>TC>TT) and lncAPP (CC<TC<TT) according to GTEx. **e**, Expression of lncAPP and mRNA stratified by rs2154481 allele C carriers or non-carriers in AD cases and controls respectively. Interestingly, we saw an increase in the expression of the lncAPP associated with the T allele that seems more exacerbated in the patients than in the controls. If so, the protective C allele (rs2154481) would also be associated with a decrease in the expression of the lncAPP, thus being able to modify the final expression of APP. In **c-e,** data are represented as boxplots where the middle line is the median, the lower and upper hinges correspond to the first and third quartiles, the whiskers extend to the hinge to the inter-quartile range (IQR), while data beyond the end of the whiskers are outlying points that are plotted individually according to the manual of ggplot2 package in R. . *12*

Supplementary Figure 5. **Expression for CHRNE eQTL. a**, Differential tissue expression for CHRNE eQTL according to GTEX. Data are presented as Normalized effect size (NES) with 95% CI. **b-c**, Expression of the CHRNE transcript in the brain according to BrainSeq and GTEx respectively. Data are represented as boxplots where the middle line is the median, the lower and upper hinges correspond to the first and third quartiles, the whiskers extend to the hinge to the inter-quartile range (IQR), while data beyond the end of the whiskers are outlying points that are plotted individually according to the manual of ggplot2 package in R. *13*

Supplementary Data

Supplementary Data 1. Description of the data sets used for meta-analysis and replication.
Supplementary Data 2. Replication of the SNPs selected with p-value $<10^{-05}$ within the new loci annotated.
Supplementary Data 3. Results of significant genome-wide association meta-analysis of AD by Proxy and case/control status by cohorts.
Supplementary Data 4. The genetic landscape of late-onset Alzheimer's disease.
Supplementary Data 5. Variants included to perform the polygenic risk score.
Supplementary Data 6. 50 risk tiles of the PRS.
Supplementary Data 7. Association of the continuous AD-PRS within each *APOE* groups using logistic regression models adjusted for four population ancestry components.
Supplementary Data 8. Risk extremes comparisons between the *APOE* ε4ε4 highest PRS risk tile to the APOE ε2ε2/ε2ε3 lowest PRS risk tile.
Supplementary Data 9. COX regression model on age at onset based on case-only in GR@ACE/DEGESCO cohort.
Supplementary Data 10. Description of datasets within DEGESCO/GR@ACE consortium.
Supplementary Data 11. Quality control for GR@ACE discovery dataset.
Supplementary Data 12. Samples within EADB France node.
Supplementary Data 13. Conditional analysis on chromosome 16, *PLCG2* region.
Supplementary Data 14. Conditional analysis on chromosome 17, *MINK1/CHRNE* and *SCIMP* region.
Supplementary Data 15. Gene-set association results for curated gene-sets.
Supplementary Data 16. Summary statistics and functional annotation for SNPs reaching genome-wide significance in the GR@ACE, IGAP and UK Biobank meta-analysis for Alzheimer's disease.
Supplementary Data 17. Genes significantly associated with AD in gene-based association tests.
Supplementary Data 18. Genes significantly associated with AD implicated by positional mapping, eQTL, chromatin interaction and gene-based analysis.

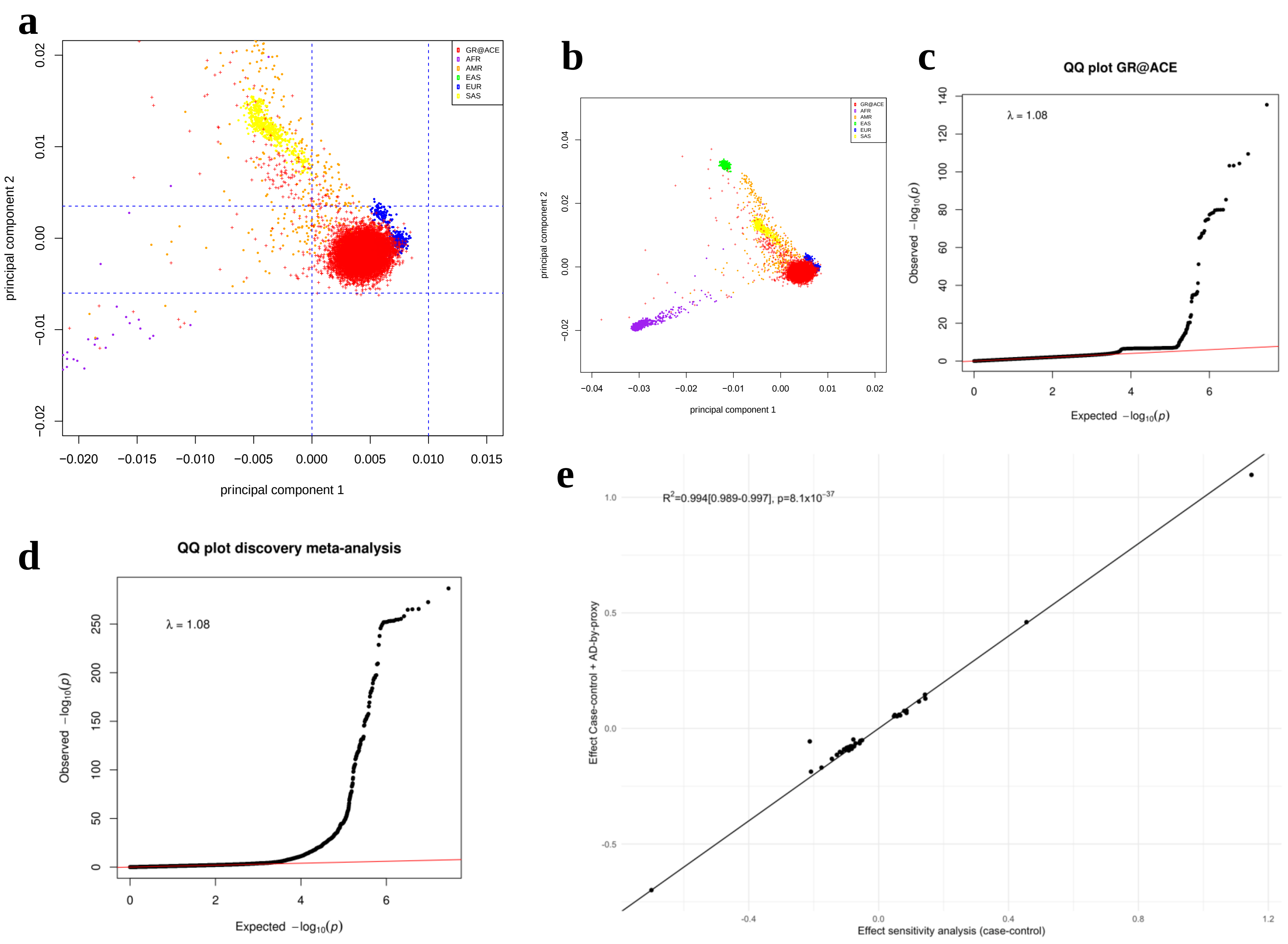

Supplementary Figure 1. **Genome-wide association study.**

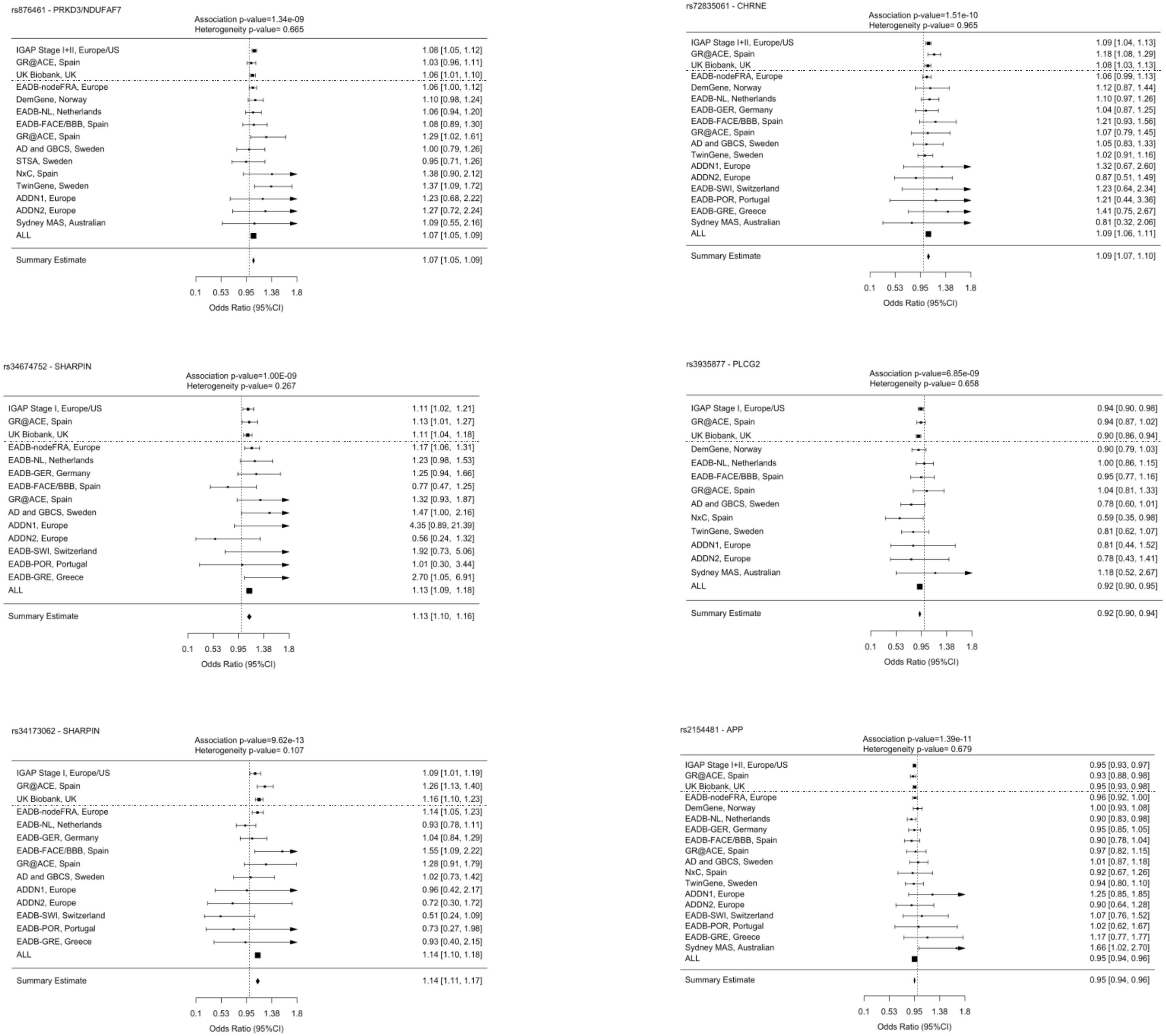

Supplementary Figure 2. **Forest plots for the six novel signals identified in overall meta-analysis.**

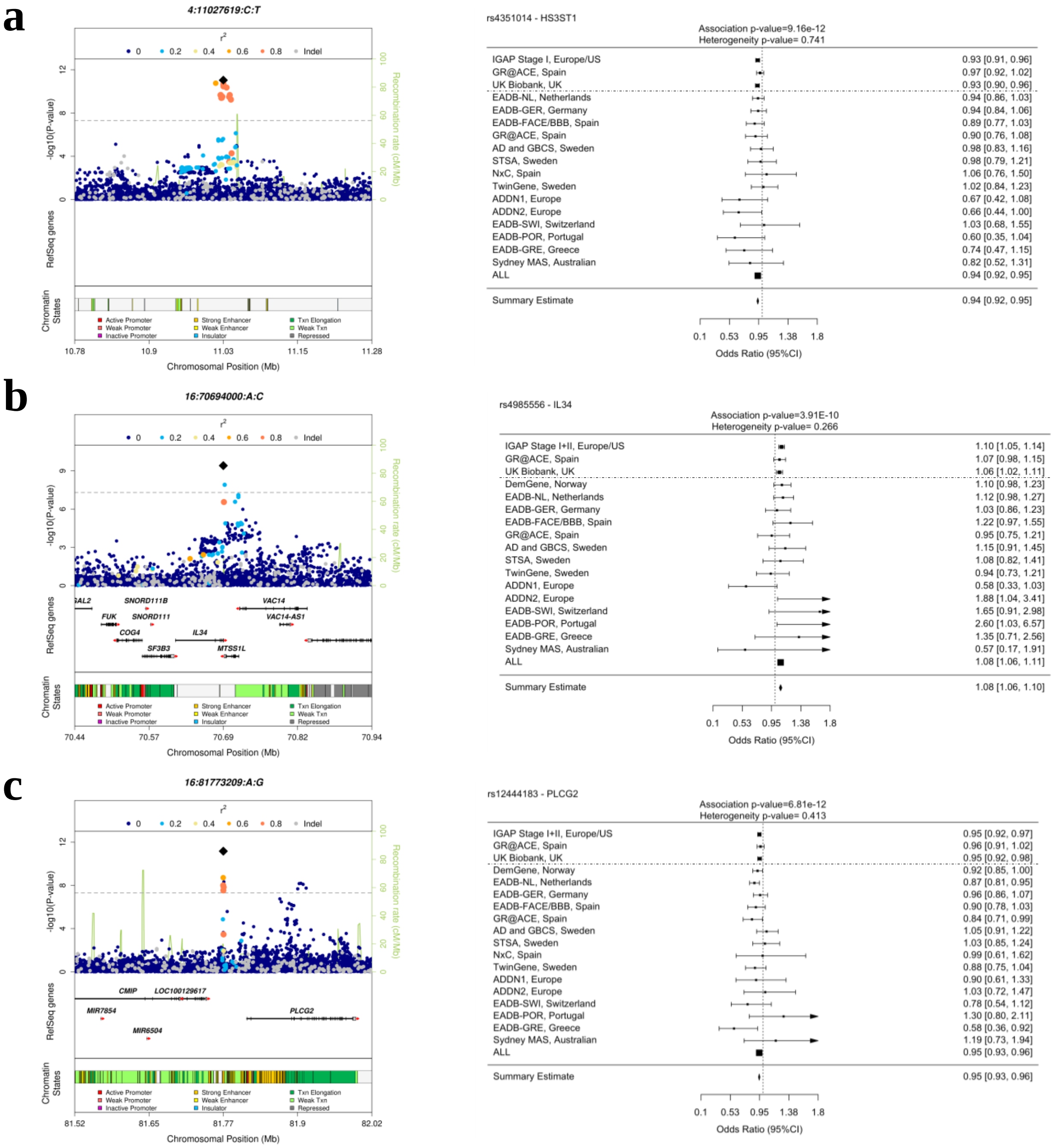

Supplementary Figure 3. **LocusZoom and forest plots: strengthened evidence of association with AD for three additional genomic loci.**

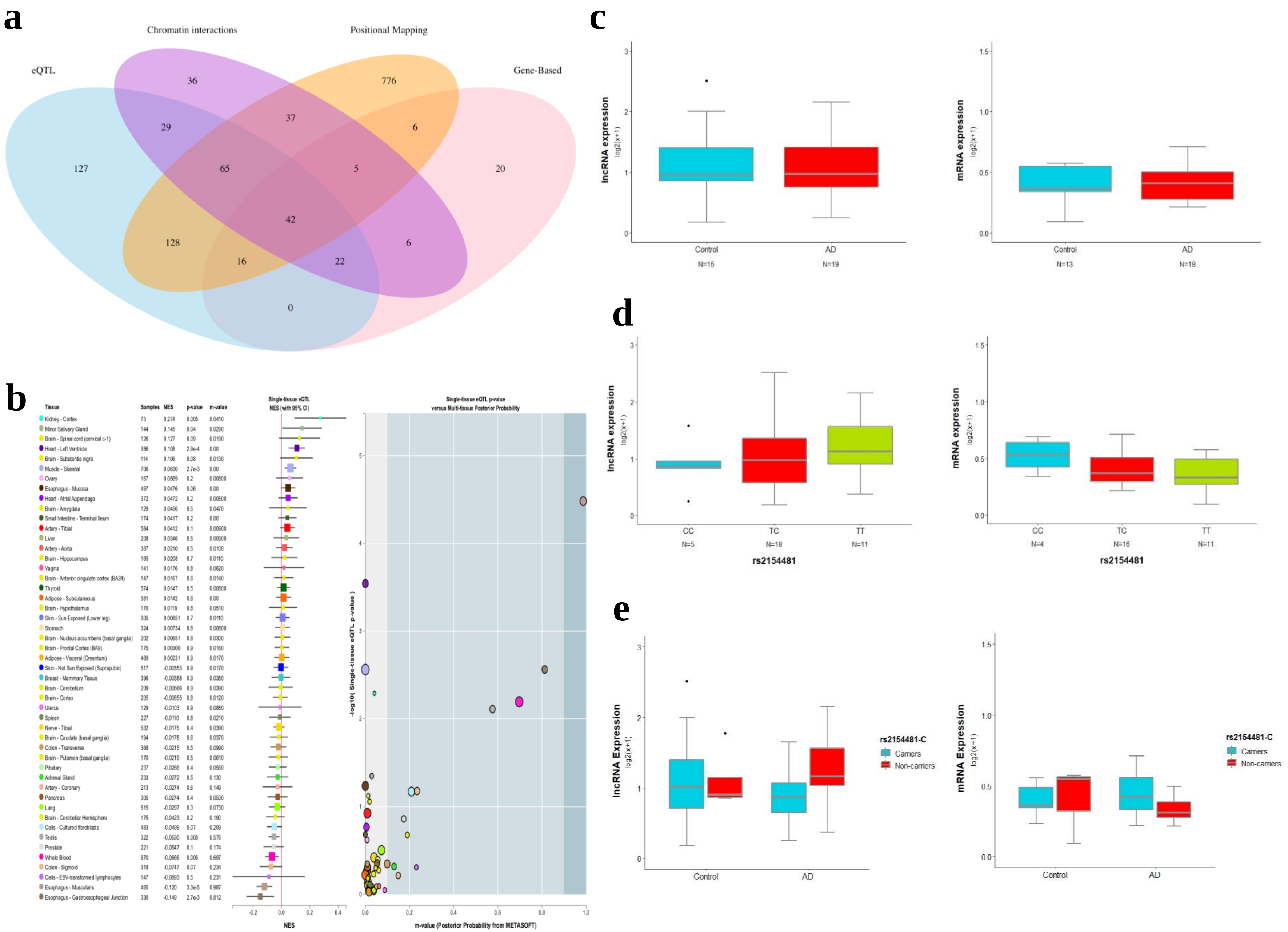

Supplementary Figure 4. Functional analysis.

a

Tissue	Samples	NES	p-value	m-value
Minor Salivary Gland	144	1.72	2.3e-13	1.00
Nerve - Tibial	532	1.48	6.4e-57	1.00
Artery - Tibial	584	1.45	4.8e-75	1.00
Cells - Cultured fibroblasts	483	1.45	2.5e-83	1.00
Colon - Sigmoid	318	1.44	3.5e-29	1.00
Adipose - Subcutaneous	581	1.39	2.7e-73	1.00
Breast - Mammary Tissue	396	1.37	2.4e-42	1.00
Esophagus - Muscularis	465	1.35	2.2e-55	1.00
Colon - Transverse	368	1.34	5.5e-34	1.00
Artery - Aorta	387	1.34	6.0e-45	1.00
Thyroid	574	1.31	1.0e-62	1.00
Artery - Coronary	213	1.29	7.7e-24	1.00
Esophagus - Mucosa	497	1.29	2.9e-50	1.00
Esophagus - Gastroesophageal Junction	330	1.27	4.5e-29	1.00
Stomach	324	1.20	3.4e-33	1.00
Adipose - Visceral (Omentum)	469	1.19	1.8e-47	1.00
Lung	515	1.17	9.0e-63	1.00
Skin - Not Sun Exposed (Suprapubic)	517	1.11	8.9e-68	1.00
Uterus	129	1.10	9.6e-7	1.00
Skin - Sun Exposed (Lower leg)	605	1.10	4.3e-83	1.00
Small Intestine - Terminal Ileum	174	1.07	1.5e-14	1.00
Brain - Substantia nigra	114	1.06	3.4e-9	1.00
Brain - Frontal Cortex (BA9)	175	1.05	2.1e-13	1.00
Vagina	141	1.04	8.3e-8	1.00
Heart - Left Ventricle	386	1.03	5.2e-30	1.00
Cells - EBV-transformed lymphocytes	147	1.03	2.2e-11	1.00
Kidney - Cortex	73	1.02	2.4e-5	1.00
Spleen	227	1.02	1.5e-10	1.00
Brain - Cortex	205	1.01	3.3e-16	1.00
Adrenal Gland	233	0.960	3.7e-19	1.00
Brain - Hypothalamus	170	0.958	1.3e-12	1.00
Brain - Putamen (basal ganglia)	170	0.930	1.6e-12	1.00
Brain - Anterior cingulate cortex (BA24)	147	0.903	6.9e-10	1.00
Brain - Nucleus accumbens (basal ganglia)	202	0.872	2.1e-11	1.00
Brain - Hippocampus	165	0.860	4.2e-10	1.00
Brain - Amygdala	129	0.831	2.5e-9	1.00
Prostate	221	0.811	2.9e-13	1.00
Brain - Spinal cord (cervical c-1)	126	0.774	2.5e-6	1.00
Whole Blood	670	0.765	4.4e-37	1.00
Ovary	167	0.751	2.1e-6	1.00
Brain - Caudate (basal ganglia)	194	0.749	3.3e-10	1.00
Brain - Cerebellum	209	0.640	1.0e-7	1.00
Brain - Cerebellar Hemisphere	175	0.621	1.1e-6	0.976
Muscle - Skeletal	706	0.601	7.8e-18	1.00
Pituitary	237	0.0901	0.3	0.00
Pancreas	305	-0.00299	1	0.00
Testis	322	-0.0106	0.9	0.00
Liver	208	-0.122	0.4	0.00
Heart - Atrial Appendage	372	-0.232	3.6e-3	0.00

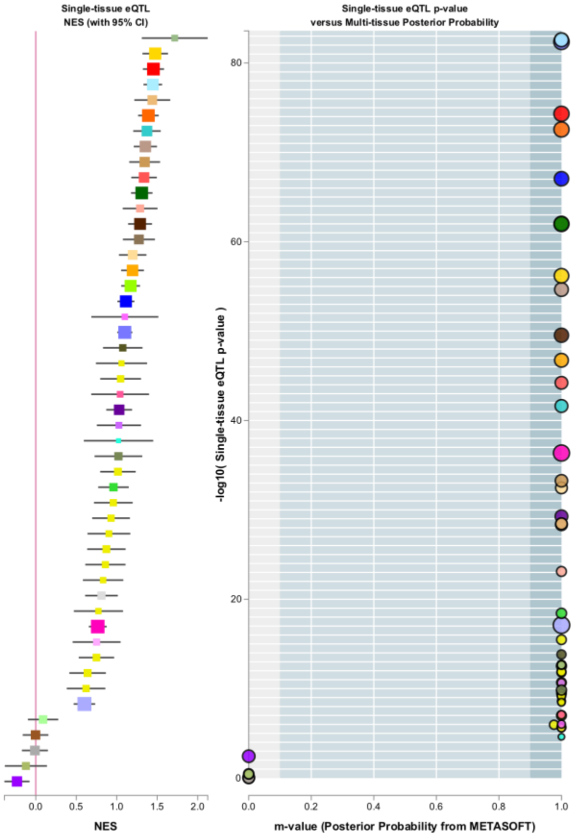

b

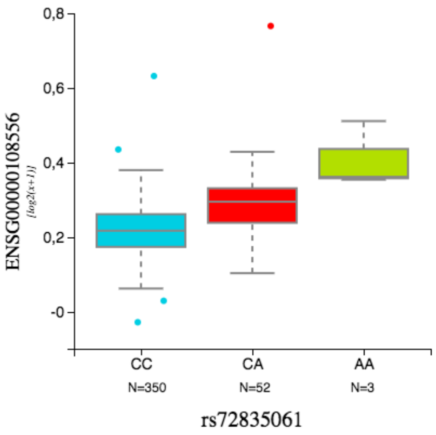

c

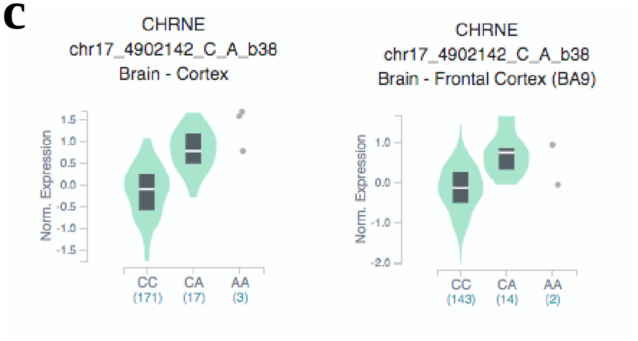

Supplementary Figure 5. Expression for CHRNE eQTL.

Supplementary Note

Additional support for EADB cohorts was provided by: Italian Ministry of Health (Ricerca Corrente); Ministero dell'Istruzione, dell'Università e della Ricerca–MIUR project "Dipartimenti di Eccellenza 2018–2022" to Department of Neuroscience "Rita Levi Montalcini", University of Torino (IR), and AIRAlzh Onlus-ANCC-COOP (SB); Partly supported by "Ministero della Salute", I.R.C.C.S. Research Program, Ricerca Corrente 2018-2020, Linea n. 2 "Meccanismi genetici, predizione e terapie innovative delle malattie complesse" and by the "5 x 1000" voluntary contribution to the Fondazione I.R.C.C.S. Ospedale "Casa Sollievo della Sofferenza"; and RF-2018-12366665, Fondi per la ricerca 2019 (Sandro Sorbi). Copenhagen General Population Study (CGPS): We thank staff and participants of the CGPS for their important contributions. Karolinska Institutet AD cohort: Dr. Graff and co-authors of the Karolinska Institutet AD cohort report grants from Swedish Research Council (VR) 2015-02926, 2018-02754, 2015-06799, Swedish Alzheimer Foundation, Stockholm County Council ALF and resarch school, Karolinska Institutet StratNeuro, Swedish Demensfonden, and Swedish brain foundation, during the conduct of the study. ADGEN: This work was supported by Academy of Finland (grant numbers 307866); Sigrid Jusélius Foundation; the Strategic Neuroscience Funding of the University of Eastern Finland; EADB project in the JPNDCO-FUND program (grant number 301220). CBAS: Supported by the project no. LQ1605 from the National Program of Sustainability II (MEYS CR), Supported by Ministry of Health of the Czech Republic, grant nr. NV19-04-00270 (All rights reserved), Grant Agency of Charles University Grants No. 693018 and 654217; the Ministry of Health, Czech Republic—conceptual development of research organization, University Hospital Motol, Prague, Czech Republic Grant No. 00064203; the Czech Ministry of Health Project AZV Grant No. 16—27611A; and Institutional Support of Excellence 2. LF UK Grant No. 699012. CNRMAJ-Rouen: This study received fundings from the Centre National de Référence Malades Alzheimer Jeunes (CNRMAJ). The Finnish Geriatric Intervention Study for the Prevention of Cognitive Impairment and Disability (FINGER) data collection was supported by grants from the Academy of Finland, La Carita Foundation, Juho Vainio Foundation, Novo Nordisk Foundation, Finnish Social Insurance Institution, Ministry of Education and Culture Research Grants, Yrjö Jahnsson Foundation, Finnish Cultural Foundation South Osthrobothnia Regional Fund, and EVO/State Research Funding grants of University Hospitals of Kuopio, Oulu and Turku, Seinäjoki Central Hospital and Oulu City Hospital, Alzheimer's Research & Prevention Foundation USA, AXA Research Fund, Knut and Alice Wallenberg Foundation Sweden, Center for Innovative Medicine (CIMED) at Karolinska Institutet Sweden, and Stiftelsen Stockholms sjukhem Sweden. FINGER cohort genotyping was funded by EADB project in the JPND CO-FUND (grant number 301220). Research on the Belgian EADB cohort is funded in part by the Alzheimer Research Foundation (SAO-FRA), The Research Foundation Flanders (FWO), and the University of Antwerp Research Fund. FK is supported by a BOF DOCPRO fellowship of the University of Antwerp Research Fund. SNAC-K is financially supported by the Swedish Ministry of Health and Social Affairs, the participating County Councils and Municipalities, and the Swedish Research Council. BDR Bristol: We would like to thank the South West Dementia Brain Bank (SWDBB) for providing brain tissue for this study. The SWDBB is part of the Brains for Dementia Research programme, jointly funded by Alzheimer's Research UK and Alzheimer's Society and is supported by BRACE (Bristol Research into Alzheimer's and Care of the Elderly) and the Medical Research Council. BDR Manchester: We would like to thank the Manchester Brain Bankfor providing brain tissue for this study. The Manchester Brain Bank is part of the Brains for Dementia Research programme, jointly funded by Alzheimer's Research UK and Alzheimer's Society. BDR KCL: Human post-mortem tissue was provided by the London Neurodegenerative Diseases Brain Bank which receives funding from the UK Medical Research Council and as part of the Brains for Dementia Research programme, jointly funded by Alzheimer's Research UK and the Alzheimer's Society. The CFAS Wales study was funded by the ESRC (RES-060-25-0060) and HEFCW as 'Maintaining function and well-being in later life: a longitudinal cohort study', (Principal Investigators: R.T Woods, L.Clare, G.Windle, V. Burholt, J. Philips, C. Brayne, C. McCracken, K. Bennett, F. Matthews). We are grateful to the NISCHR Clinical Research Centre for their assistance in tracing participants and in interviewing and in collecting blood samples, and to general practices in the study areas for their cooperation. MRC: We thank all individuals who participated in this study. Cardiff University was supported by the Alzheimer's Society (AS; grant RF014/164) and the Medical Research Council (MRC; grants G0801418/1, MR/K013041/1, MR/L023784/1) (R. Sims is an AS Research Fellow). Cardiff University was also supported by the European Joint Programme for Neurodegenerative Disease (JPND; grant MR/L501517/1), Alzheimer's Research UK (ARUK; grant ARUK-PG2014-1), the Welsh Assembly Government (grant SGR544:CADR), Brain's for dementia Research and a donation from the Moondance Charitable Foundation. Cardiff University acknowledges the support of the UK Dementia Research Institute, of which J. Williams is an associate director. Cambridge University acknowledges support from the MRC. Patient recruitment for the MRC Prion Unit/UCL Department of Neurodegenerative Disease

collection was supported by the UCLH/UCL Biomedical Centre and NIHR Queen Square Dementia Biomedical Research Unit. The University of Southampton acknowledges support from the AS. King's College London was supported by the NIHR Biomedical Research Centre for Mental Health and the Biomedical Research Unit for Dementia at the South London and Maudsley NHS Foundation Trust and by King's College London and the MRC. ARUK and the Big Lottery Fund provided support to Nottingham University. The MEMENTO cohort is funded by the Fondation Plan Alzheimer (Alzheimer Plan 2008–2012), and the French Ministry of research (MESRI, DGRI) through the Plan Maladies Neurodégénératives (2014-2019). This work was also supported by the following: CIC 1401-EC, Bordeaux University Hospital (CHU Bordeaux, sponsor of the cohort), Inserm and the University of Bordeaux. Memento GWAS was funded through JPND grant. German Study on Ageing, Cognition and Dementia in Primary Care Patients (AgeCoDe): This study/publication is part of the German Research Network on Dementia (KND), the German Research Network on Degenerative Dementia (KNDD; German Study on Ageing, Cognition and Dementia in Primary Care Patients; AgeCoDe), and the Health Service Research Initiative (Study on Needs, health service use, costs and health-related quality of life in a large sample of oldestold primary care patients (85+; AgeQualiDe)) and was funded by the German Federal Ministry of Education and Research (grants KND: 01GI0102, 01GI0420, 01GI0422, 01GI0423, 01GI0429, 01GI0431, 01GI0433, 01GI0434; grants KNDD: 01GI0710, 01GI0711, 01GI0712, 01GI0713, 01GI0714, 01GI0715, 01GI0716; grants Health Service Research Initiative: 01GY1322A, 01GY1322B, 01GY1322C, 01GY1322D, 01GY1322E, 01GY1322F, 01GY1322G). VITA study: The support of the Ludwig Boltzmann Society and the AFI Germany have supported the VITA study. The former VITA study group should be acknowledged: W. Danielczyk, G. Gatterer, K. Jellinger, S. Jugwirth, K.H. Tragl, S. Zehetmayer. Vogel Study: This work was financed by a research grant of the ‘‘Vogelstiftung Dr. Eckernkamp’’. HELIAD study: This study was supported by the grants: IIRG-09-133014 from the Alzheimer’s Association, 189 10276/8/9/2011 from the ESPA-EU program Excellence Grant (ARISTEIA) and the ΔΥ2β/οικ.51657/14.4.2009 of the Ministry for Health and Social Solidarity (Greece). Biobank Department of Psychiatry, UMG: Prof. Jens Wiltfang is supported by an Ilídio Pinho professorship and iBiMED (UID/BIM/04501/2013), and FCT project PTDC/DTP_PIC/5587/2014 at the University of Aveiro, Portugal. Lausanne study: This work was supported by grants from the Swiss National Research Foundation (SNF 320030_141179). PAGES study: Harald Hampel is an employee of Eisai Inc. During part of this work he was supported by the AXA Research Fund, the “Fondation partenariale Sorbonne Université” and the “Fondation pour la Recherche sur Alzheimer”, Paris, France. Mannheim, Germany Biobank: Department of geriatric Psychiatry, Central Institute for Mental Health, Mannheim, University of Heidelberg, Germany. Genotyping for the Swedish Twin Studies of Aging was supported by NIH/NIA grant R01 AG037985. Genotyping in TwinGene was supported by NIH/NIDDK U01 DK066134. WvdF is recipient of Joint Programming for Neurodegenerative Diseases (JPND) grants PERADES (ANR-13-JPRF-0001) and EADB (733051061). Gothenburg Birth Cohort (GBC) Studies: We would like to thank UCL Genomics for performing the genotyping analyses. The studies were supported by The Stena Foundation, The Swedish Research Council (2015-02830, 2013-8717), The Swedish Research Council for Health, Working Life and Wellfare (2013-1202, 2005-0762, 2008-1210, 2013-2300, 2013- 2496, 2013-0475), The Brain Foundation, Sahlgrenska University Hospital (ALF), The Alzheimer’s Association (IIRG-03-6168), The Alzheimer’s Association Zenith Award (ZEN-01-3151), Eivind och Elsa K: son Sylvans Stiftelse, The Swedish Alzheimer Foundation. Clinical AD, Sweden: We would like to thank UCL Genomics for performing the genotyping analyses. Barcelona Brain Biobank: Brain Donors of the Neurological Tissue Bank of the Biobanc-Hospital Clinic-IDIBAPS and their families for their generosity. Hospital Clínic de Barcelona Spanish Ministry of Economy and Competitiveness-Instituto de Salud Carlos III and Fondo Europeo de Desarrollo Regional (FEDER), Unión Europea, “Una manera de hacer Europa” grants (PI16/0235 to Dr. R. Sánchez-Valle and PI17/00670 to Dr. A.Antonell). AA is funded by Departament de Salut de la Generalitat de Catalunya, PERIS 2016-2020 (SLT002/16/00329). Sydney Memory and Ageing Study (Sydney MAS): We gratefully acknowledge and thank the following for their contributions to Sydney MAS: participants, their supporters and the Sydney MAS Research Team (current and former staff and students). Funding was awarded from the Australian National Health and Medical Research Council (NHMRC) Program Grants (350833, 568969, 109308). The AddNeuroMed data are from a public-private partnership supported by EFPIA companies and SMEs as part of InnoMed (Innovative Medicines in Europe), an integrated project funded by the European Union of the Sixth Framework program priority FP6-2004-LIFESCIHEALTH-5. Clinical leads responsible for data collection are Iwona K1oszewska (Lodz), Simon Lovestone (London), Patrizia Mecocci (Perugia), Hilkka Soininen (Kuopio), Magda Tsolaki (Thessaloniki), and Bruno Vellas (Toulouse). Imaging leads are Andy Simmons (London), Lars-Olad Wahlund (Stockholm), and Christian Spenger (Zurich). Bioinformatics leads are Richard Dobson (London) and Stephen Newhouse (London). The Neocodex-Murcia study was funded by the Fundación Alzheimur (Murcia), the Ministerio de Educación y Ciencia (Gobierno de España), Corporación Tecnológica de Andalucía, Agencia IDEA

(Consejería de Innovación, Junta de Andalucía), the Diabetes Research Laboratory, and the Biomedical Research Foundation. University Hospital Clínico San Carlos has been supported by CIBER de Diabetes y Enfermedades Metabólicas Asociadas (CIBERDEM); CIBERDEM is an ISCIII Project. Oviedo: This work was partly supported by Grant from Fondo de Investigaciones Sanitarias-Fondos FEDER EuropeanUnion to Victoria Alvarez PI15/00878. Pascual Sánchez-Juan is supported by CIBERNED and Carlos III Institute of Health, Spain (PI08/0139, PI12/02288, and PI16/01652), jointly funded by Fondo Europeo de Desarrollo Regional (FEDER), Unión Europea, "Una manera de hacer Europa". Project MinE: The ProjectMinE study was supported by the ALS Foundation Netherlands and the MND association (UK) (Project MinE, www.projectmine.com). The SPIN cohort: We are indebted to patients and their families for their participation in the "Sant Pau Initiative on Neurodegeneration cohort", at the Sant Pau Hospital (Barcelona). This is a multimodal research cohort for biomarker discovery and validation that is partially funded by Generalitat de Catalunya (2017 SGR 547 to JC), as well as from the Institute of Health Carlos III-Subdirección General de Evaluación and the Fondo Europeo de Desarrollo Regional (FEDER- "Una manera de Hacer Europa") (grants PI11/02526, PI14/01126, and PI17/01019 to JF; PI17/01895 to AL), and the Centro de Investigación Biomédica en Red Enfermedades Neurodegenerativas programme (Program 1, Alzheimer Disease to AL). We would also like to thank the Fundació Bancària Obra Social La Caixa (DABNI project) to JF and AL; and Fundación BBVA (to AL), for their support in funding this follow-up study. Adolfo López de Munain is supported by Fundación Salud 2000 (PI2013156) and Diputación Foral de Gipuzkoa (Exp.114/17). The Vascular Cognitive Impairment Genetics Consortium (VCIGC) was initially set up 2010-2013 by The Dementia Research Group, University of Bristol, to undertake pilot investigations into the genetic basis of forms of Vascular Cognitive Impairment (VCI)[47]. The resultant collaborative collection consists of several thousand cases (vascular dementia, post-stroke dementia or mixed vascular dementia and Alzheimer's disease) and controls from a number of UK and International Centres with an intended use for further research into the genetic aetiology of VCI. DNA from post-mortem neuropathologically diagnosed control cases were utilised from three UK Brain Bank cohorts (Bristol, Newcastle, Oxford) that are part of the VCIGC. All contributing brain banks have local ethics approval. The South West Dementia Brain Bank (SWDBB) provided DNA samples to the University of Bristol. The SWDBB is part of the Brains for Dementia Research programme, jointly funded by Alzheimer's Research UK and Alzheimer's Society and is supported by BRACE (Bristol Research into Alzheimer's and Care of the Elderly) and the Medical Research Council. Tissue was provided by The Newcastle Brain Tissue Resource which is funded in part by: a grant from the UK Medical Research Council (G0400074), by NIHR Newcastle Biomedical Research Centre and Unit awarded to the Newcastle upon Tyne NHS Foundation Trust and Newcastle University, and as part of the Brains for Dementia Research Programme, jointly funded by Alzheimer's Research UK and Alzheimer's Society. Oxford samples are part of the NIHR Oxford Biomedical Research Centre supported Oxford Project to Investigate Memory and Ageing (OPTIMA) study. We acknowledge the Oxford Brain Bank, supported by the Medical Research Council (MRC), Brains for Dementia Research (BDR) (Alzheimer Society and Alzheimer Research UK), Autistica UK and the NIHR Oxford Biomedical Research Centre.

Supplementary References

1. Watanabe, K., Taskesen, E., van Bochoven, A. & Posthuma, D. Functional mapping and annotation of genetic associations with FUMA. *Nat. Commun.* (2017) doi:10.1038/s41467-017-01261-5.
2. Jansen, I. E. *et al.* Genome-wide meta-analysis identifies new loci and functional pathways influencing Alzheimer's disease risk. *Nat. Genet.* **51**, 404–413 (2019).
3. Wang, K., Li, M. & Hakonarson, H. ANNOVAR: functional annotation of genetic variants from high-throughput sequencing data. *Nucleic Acids Res.* **38**, e164–e164 (2010).
4. Kircher, M. *et al.* A general framework for estimating the relative pathogenicity of human genetic variants. *Nat. Genet.* **46**, 310–5 (2014).
5. Boyle, A. P. *et al.* Annotation of functional variation in personal genomes using RegulomeDB. *Genome Res.* **22**, 1790–1797 (2012).
6. Ernst, J. & Kellis, M. ChromHMM: automating chromatin-state discovery and characterization. *Nat. Methods* **9**, 215–216 (2012).
7. Lek, M. *et al.* Analysis of protein-coding genetic variation in 60,706 humans. *Nature* **536**, 285–291 (2016).
8. Petrovski, S. *et al.* The Intolerance of Regulatory Sequence to Genetic Variation Predicts Gene Dosage Sensitivity. *PLOS Genet.* **11**, e1005492 (2015).
9. The GTEx Consortium *et al.* The Genotype-Tissue Expression (GTEx) pilot analysis: Multitissue gene regulation in humans. *Science (80-.).* (2015) doi:10.1126/science.1262110.
10. Westra, H.-J. *et al.* Systematic identification of trans eQTLs as putative drivers of known disease associations. *Nat. Genet.* **45**, 1238–1243 (2013).
11. Zhernakova, D. V *et al.* Identification of context-dependent expression quantitative trait loci in whole blood. *Nat. Genet.* **49**, 139–145 (2017).
12. de Leeuw, C. A., Mooij, J. M., Heskes, T. & Posthuma, D. MAGMA: Generalized Gene-Set Analysis of

GWAS Data. *PLoS Comput. Biol.* (2015) doi:10.1371/journal.pcbi.1004219.
13. BrainSeq: A Human Brain Genomics Consortium. Electronic address: drweinberger@libd.org, C. R. *et al.* BrainSeq: Neurogenomics to Drive Novel Target Discovery for Neuropsychiatric Disorders. *Neuron* **88**, 1078–1083 (2015).
14. De Roeck, A. *et al.* An intronic VNTR affects splicing of ABCA7 and increases risk of Alzheimer's disease. *Acta Neuropathol.* **135**, 827–837 (2018).
15. Sheardova, K. *et al.* Czech Brain Aging Study (CBAS): prospective multicentre cohort study on risk and protective factors for dementia in the Czech Republic. *BMJ Open* **9**, (2019).
16. Steinberg, S. *et al.* Loss-of-function variants in ABCA7 confer risk of Alzheimer's disease. *Nat. Genet.* **47**, 445–447 (2015).
17. Ngandu, T. *et al.* A 2 year multidomain intervention of diet, exercise, cognitive training, and vascular risk monitoring versus control to prevent cognitive decline in at-risk elderly people (FINGER): a randomised controlled trial. *Lancet* **385**, 2255–2263 (2015).
18. Hanon, O. *et al.* Plasma amyloid levels within the Alzheimer's process and correlations with central biomarkers. *Alzheimers. Dement.* **14**, 858–868 (2018).
19. Dufouil, C. *et al.* Cognitive and imaging markers in non-demented subjects attending a memory clinic: study design and baseline findings of the MEMENTO cohort. *Alzheimers. Res. Ther.* **9**, 67 (2017).
20. Nicolas, G. *et al.* Screening of dementia genes by whole-exome sequencing in early-onset Alzheimer disease: input and lessons. *Eur. J. Hum. Genet.* **24**, 710–6 (2016).
21. Casati, M. *et al.* Vitamin E and Alzheimer's disease: the mediating role of cellular aging. *Aging Clin. Exp. Res.* (2019) doi:10.1007/s40520-019-01209-3.
22. Casati, M. *et al.* Increased expression of TREM2 in peripheral cells from mild cognitive impairment patients who progress into Alzheimer's disease. *Eur. J. Neurol.* **25**, 805–810 (2018).
23. Ferri, E. *et al.* Apolipoprotein E gene in physiological and pathological aging. *Mech. Ageing Dev.* **178**, 41–45 (2019).
24. Alcolea, D. *et al.* The Sant Pau Initiative on Neurodegeneration (SPIN) cohort: A data set for biomarker discovery and validation in neurodegenerative disorders. *Alzheimer's Dement. (New York, N. Y.)* **5**, 597–609 (2019).
25. Kornhuber, J. *et al.* Early and Differential Diagnosis of Dementia and Mild Cognitive Impairment. *Dement. Geriatr. Cogn. Disord.* **27**, 404–417 (2009).
26. Luck, T. *et al.* Mild Cognitive Impairment in General Practice: Age-Specific Prevalence and Correlate Results from the German Study on Ageing, Cognition and Dementia in Primary Care Patients (AgeCoDe). *Dement. Geriatr. Cogn. Disord.* **24**, 307–316 (2007).
27. Grau-Rivera, O. *et al.* Clinicopathological Correlations and Concomitant Pathologies in Rapidly Progressive Dementia: A Brain Bank Series. *Neurodegener. Dis.* (2015) doi:10.1159/000439251.
28. Holstege, H. *et al.* The 100-plus Study of cognitively healthy centenarians: rationale, design and cohort description. *Eur. J. Epidemiol.* (2018) doi:10.1007/s10654-018-0451-3.
29. Huisman, M. *et al.* Cohort Profile: The Longitudinal Aging Study Amsterdam. *Int. J. Epidemiol.* **40**, 868–876 (2011).
30. Hoogendijk, E. O. *et al.* The Longitudinal Aging Study Amsterdam: cohort update 2016 and major findings. *Eur. J. Epidemiol.* **31**, 927–945 (2016).
31. van der Flier, W. M. *et al.* Optimizing patient care and research: the Amsterdam Dementia Cohort. *J. Alzheimer's Dis. JAD* **41**, 313–327 (2014).
32. Van Der Flier, W. M. & Scheltens, P. Amsterdam dementia cohort: Performing research to optimize care. *Journal of Alzheimer's Disease* (2018) doi:10.3233/JAD-170850.
33. Sachdev, P. S. *et al.* The Sydney Memory and Ageing Study (MAS): methodology and baseline medical and neuropsychiatric characteristics of an elderly epidemiological non-demented cohort of Australians aged 70–90 years. *Int. Psychogeriatrics* **22**, 1248–1264 (2010).
34. Zetterberg, H. *et al.* Clinical proteomics in neurodegenerative disorders. *Acta Neurologica Scandinavica* (2008) doi:10.1111/j.1600-0404.2007.00985.x.
35. Bengtsson, C. *et al.* THE STUDY OF WOMEN IN GOTHENBURG 1968-1969 -A POPULATION STUDY. *Acta Med. Scand.* **193**, 311–318 (2009).
36. Skoog, I. *et al.* A 9-Year Prospective Population-Based Study on the Association Between the APOE*E4 Allele and Late-Life Depression in Sweden. *Biol. Psychiatry* **78**, 730–736 (2015).
37. Zettergren, A. *et al.* The ACE Gene Is Associated with Late-Life Major Depression and Age at Dementia Onset in a Population-Based Cohort. *Am. J. Geriatr. Psychiatry* (2017) doi:10.1016/j.jagp.2016.06.009.
38. Skoog, I. *et al.* Decreasing prevalence of dementia in 85-year olds examined 22 years apart: the influence of education and stroke. *Sci. Rep.* **7**, 6136 (2017).
39. Skoog, J. *et al.* A Longitudinal Study of the Mini-Mental State Examination in Late Nonagenarians and Its Relationship with Dementia, Mortality, and Education. *J. Am. Geriatr. Soc.* **65**, 1296–1300 (2017).
40. Gayán, J. *et al.* Genetic structure of the Spanish population. *BMC Genomics* **11**, 326 (2010).
41. Antúnez, C. *et al.* The membrane-spanning 4-domains, subfamily A (MS4A) gene cluster contains a common variant associated with Alzheimer's disease. *Genome Med.* **3**, 33 (2011).
42. McKhann, G. *et al.* Clinical diagnosis of Alzheimer's disease: Report of the NINCDS-ADRDA Work Group* under the auspices of Department of Health and Human Services Task Force on Alzheimer's Disease. *Neurology* **34**, 939–939 (1984).
43. Proitsi, P. *et al.* Genetic predisposition to increased blood cholesterol and triglyceride lipid levels and risk of

Alzheimer disease: a Mendelian randomization analysis. *PLoS Med.* **11**, e1001713 (2014).
44. Lourdusamy, A. *et al.* Identification of *cis*- regulatory variation influencing protein abundance levels in human plasma. *Hum. Mol. Genet.* **21**, 3719–3726 (2012).
45. Furney, S. J. *et al.* Genome-wide association with MRI atrophy measures as a quantitative trait locus for Alzheimer's disease. *Mol. Psychiatry* **16**, 1130–8 (2011).
46. Alzheimers Disease - Community Portal - syn2790911. https://www.synapse.org/#!Synapse:syn2790911/wiki/70465.
47. Skrobot, O. A. *et al.* A Validation Study of Vascular Cognitive Impairment Genetics Meta-Analysis Findings in an Independent Collaborative Cohort. *J. Alzheimer's Dis.* **53**, 981–989 (2016).
