# Supplementary material for: Common variants in Alzheimer’s disease and risk stratification by polygenic risk scores
Source: Nat Commun. 2021 Jun 7;12:3417. doi: 10.1038/s41467-021-22491-8 (PMC8184987; doi:10.1038/s41467-021-22491-8)
Supplement: Supplementary file 3 — Description of Additional Supplementary Files [file 41467_2021_22491_MOESM3_ESM.pdf]

Description of Additional Supplementary Files

File Name: Supplementary Data 1
Description: Description of the data sets used for meta-analysis and replication.

File Name: Supplementary Data 2
Description: Replication of the SNPs selected with p-value $<10^{-05}$ within the new loci annotated.

File Name: Supplementary Data 3
Description: Results of significant genome-wide association meta-analysis of AD by Proxy and case/control status by cohorts.

File Name: Supplementary Data 4
Description: The genetic landscape of late-onset Alzheimer's disease

File Name: Supplementary Data 5
Description: Variants included to perform the Polygenic risk Score

File Name: Supplementary Data 6
Description: 50 risk tiles of the PRS

File Name: Supplementary Data 7
Description: Association of the continuous AD-PRS within each *APOE* groups using logistic regression models adjusted for four population ancestry components.

File Name: Supplementary Data 8
Description: Risk extremes comparisons between the *APOE* ε4ε4 highest PRS risk tile to the APOE ε2ε2/ε2ε3 lowest PRS risk tile.

File Name: Supplementary Data 9
Description: COX regression model on age at onset based on case-only in GR@ACE/DEGESCO cohort.

File Name: Supplementary Data 10
Description: Description of datasets within DEGESCO/GR@ACE consortium.

File Name: Supplementary Data 11
Description: Quality control for GR@ACE discovery dataset

File Name: Supplementary Data 12
Description: Samples within EADB France node

File Name: Supplementary Data 13
Description: Conditional analysis on chromosome 16. *PLCG2* region

File Name: Supplementary Data 14
Description: Conditional analysis on chromosome 17. *MINK1/CHRNE* and SCIMP region File

File Name: Supplementary Data 15

Description: Gene-set association results for curated gene-sets.

File Name: Supplementary Data 16
Description: Summary statistics and functional annotation for SNPs reaching genome-wide significance in the GR@ACE, IGAP and UK Biobank meta-analysis for Alzheimer's Disease.

File Name: Supplementary Data 17
Description: Genes significantly associated with AD in gene-based association tests

File Name: Supplementary Data 18
Description: Genes significantly associated with AD implicated by positional mapping, eQTL, chromatin interaction and gene-based analysis.
